# Ebola virus VP24 interacts with NP to facilitate nucleocapsid assembly and genome packaging

**DOI:** 10.1038/s41598-017-08167-8

**Published:** 2017-08-09

**Authors:** Logan Banadyga, Thomas Hoenen, Xavier Ambroggio, Eric Dunham, Allison Groseth, Hideki Ebihara

**Affiliations:** 1 Laboratory of Virology, Division of Intramural Research, National Institute of Allergy and Infectious Diseases, National Institutes of Health, Hamilton, MT 59840 USA; 2 Bioinformatics and Computational Biosciences Branch, National Institute of Allergy and Infectious Diseases, National Institutes of Health, Bethesda, MD 20892 USA; 3 Department of Molecular Medicine, Mayo Clinic, Rochester, MN 55905 USA; 4 Friedrich-Loeffler-Institut, Greifswald, Insel Riems Germany; 5 Rosetta Design Group, Burlington, VT 05401 USA

## Abstract

Ebola virus causes devastating hemorrhagic fever outbreaks for which no approved therapeutic exists. The viral nucleocapsid, which is minimally composed of the proteins NP, VP35, and VP24, represents an attractive target for drug development; however, the molecular determinants that govern the interactions and functions of these three proteins are still unknown. Through a series of mutational analyses, in combination with biochemical and bioinformatics approaches, we identified a region on VP24 that was critical for its interaction with NP. Importantly, we demonstrated that the interaction between VP24 and NP was required for both nucleocapsid assembly and genome packaging. Not only does this study underscore the critical role that these proteins play in the viral replication cycle, but it also identifies a key interaction interface on VP24 that may serve as a novel target for antiviral therapeutic intervention.

## Introduction

Ebola virus (EBOV), which belongs to the family *Filoviridae* and the order *Mononegavirales*, is the causative agent of severe hemorrhagic fever in humans and has been responsible for several large outbreaks throughout Africa, with case fatality rates reaching as high as 90%^1^. By far the largest EBOV outbreak ever recorded began in Western Africa in 2013 and resulted in over 28,000 cases and 11,000 deaths, mostly in Guinea, Liberia, and Sierra Leone, with several cases imported to Europe and the United States^2^. In addition to the devastatingly high death toll, this outbreak also ravaged the healthcare and social infrastructure of Western Africa, resulting in billions of dollars worth of economic damage and costing billions more in global response (http://www.cdc.gov/vhf/ebola/outbreaks/2014-west-africa/cost-of-ebola.html). Nevertheless, despite the significant public health threat posed by this virus, there are still no approved and fully-licensed vaccines or therapeutics to treat Ebola virus disease.

Like all filoviruses, EBOV possesses a single-stranded, negative sense RNA genome of approximately 19 kilobases that encodes seven structural proteins: the nucleoprotein (NP), virion protein 35 (VP35), VP40, the glycoprotein (GP), VP30, VP24, and the RNA-dependent RNA polymerase L^1^. The matrix protein VP40 is solely responsible for the characteristic filamentous shape of the EBOV virion, which derives its envelope from the host cell plasma membrane, while the heterodimer GP_1,2_ facilitates host cell recognition and entry^3–11^. The viral nucleocapsid is among the most complex of all mononegaviruses, consisting of NP, which binds the viral genome, as well as VP35, VP30, VP24, and L^12^. Of these five proteins, however, only NP, VP35, and VP24 are required to produce structures that are morphologically indistinguishable from nucleocapsids observed during EBOV infection^13–15^. By itself, NP oligomerizes around RNA to form helical, loosely coiled structures^16, 17^. In the presence of VP24 and VP35, these loose coils transform into a structure that is practically identical to the nucleocapsids found in infected cells^13, 14^.

Although the components of the EBOV nucleocapsid are well described, relatively little is known about how these components fit together to comprise a functional structure. In particular, the relationship between NP and VP24—which may have significant relevance to EBOV pathogenesis^18, 19^—is poorly characterized. Indeed, the molecular determinants that govern the interaction between these two proteins are unknown, as is the precise functional relevance of the interaction. Accordingly, we sought to examine the interaction between NP and VP24 and characterize its functional importance. This study is the first to identify how VP24 interacts with NP, and it underscores the critical role that these proteins play in the EBOV replication cycle. Indeed, we directly demonstrate here that the interaction between NP and VP24 is essential for nucleocapsid formation and packaging into the virion. Notably, the identification of a discrete interaction interface on VP24—with a critical functional role—may also provide a target for new and urgently needed antiviral drugs, the development of which has lagged well behind the development of vaccines.

## Results

### NP, VP35, and VP24 interact with each other

**Figure 1:**
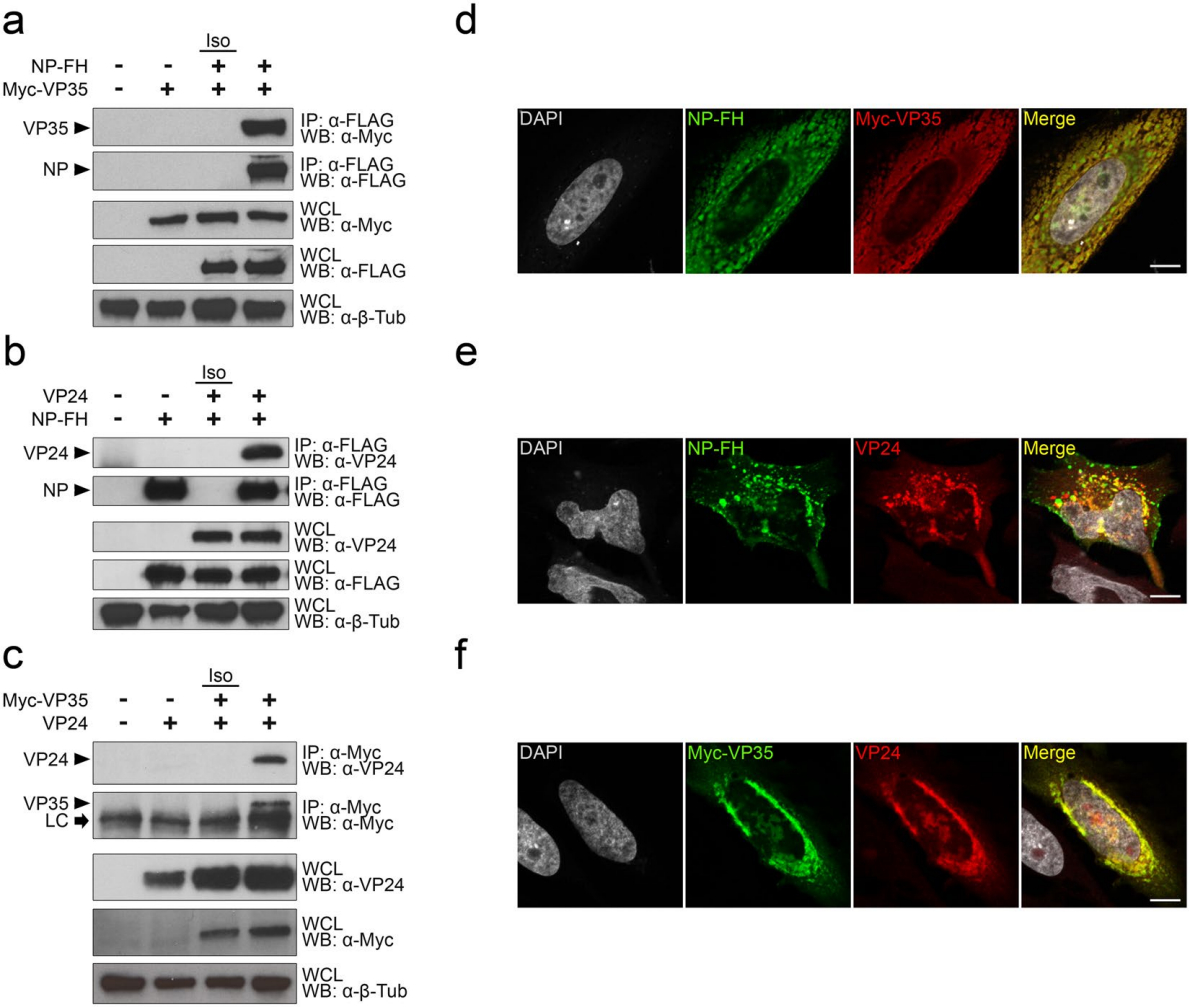
NP, VP35, and VP24 interact independently with each other. (**a**–**c**) HEK 293 cells were co-transfected with pCAGGS-NP-FH and pCAGGS-Myc-VP35 (**a**), pCAGGS-NP-FH and pCAGGS-VP24 (**b**), or pCAGGS-Myc-VP35 and pCAGGS-VP24 (**c**). Lysates were immunoprecipitated with mouse anti-FLAG (**a**,**b**), mouse anti-Myc (**c**), or isotype control (Iso; **a**,**b**) antibodies, and immunoprecipitation (IP) and whole cell lysate (WCL) fractions were subjected to Western blot (WB) with mouse anti-FLAG, mouse anti-Myc, rabbit anti-VP24, and rabbit anti-β-tubulin antibodies. The precipitated proteins in the IP fraction are labeled with arrowheads, and the light chain (LC) of the mouse anti-Myc antibody is indicated with an arrow (**a**,**b**). IP/Western blot data are representative of at least three independent experiments. Western blots were cropped for presentation; full-length blots are available in Supplementary Fig. 6. (**d**–**f**) Hela cells were co-transfected with pCAGGS-NP-FH and pCAGGS-Myc-VP35 (**d**), pCAGGS-NP-FH and pCAGGS-VP24 (**e**), or pCAGGS-Myc-VP35 and pCAGGS-VP24 (**f**). Following fixation and permeabilization, cells were stained with rabbit anti-FLAG (**d**), mouse anti-FLAG (**e**), mouse anti-Myc (**d**,**f**), and/or rabbit anti-VP24 (**e**,**f**) antibodies prior to staining with AlexaFluor 488 goat anti-mouse and AlexaFluor 546 goat anti-rabbit secondary antibodies. Coverslips were mounted with ProLong Gold Antifade Mountant containing DAPI to visualize the nuclei. White scale bars represent 10 uM.

Given the critical role played by NP, VP35, and VP24 in the formation of the EBOV nucleocapsid, we first sought to examine the interactions among these three proteins. HEK 293 cells were transfected with a combination of plasmids encoding C-terminally FLAG/HA-tagged NP (NP-FH), N-terminally Myc-tagged VP35 (Myc-VP35), and/or untagged VP24, followed by lysis in buffer containing 1% Igepal. Immunoprecipitation (IP) of cell lysates with an anti-FLAG antibody followed by Western blotting (WB) with an anti-Myc or anti-VP24 antibody demonstrated an interaction between NP and VP35 (Fig. 1a) and NP and VP24 (Fig. 1b), providing the clearest evidence so far reported that NP directly interacts with both VP35 and VP24 in the absence of any other viral protein^14, 20^. Notably, immunoprecipitation with an anti-Myc antibody revealed an interaction between VP35 and VP24, providing the first direct evidence that these two proteins interact with each other (Fig. 1c). An isotype control antibody (Iso) was used to demonstrate that each immunoprecipitation was specific to the antibody and epitope tag used and that protein complexes were not binding non-specifically to beads or antibody constant regions. Moreover, whole cell lysates (WCL) for each experiment showed similar expression levels of NP-FH, Myc-VP35, and VP24, as well as the loading control, beta-tubulin (Fig. 1a–c). Since we were interested in further characterizing the interaction between NP and VP24, we also confirmed the ability of these proteins to interact in the context of virus infection (Supplementary Fig. 1).

To provide further support for the interactions among NP, VP35, and VP24, HeLa cells were transfected with plasmids encoding these three proteins, and their co-localization was assessed by immunofluorescence. Staining with an anti-FLAG antibody revealed NP localization that overlapped with both VP35, stained with an anti-Myc antibody (Fig. 1d), and VP24, stained with an anti-VP24 antibody (Fig. 1e), as indicated by the yellow color in the merged panel. Similarly, staining with the anti-Myc and anti-VP24 antibodies demonstrated co-localization between Myc-VP35 and VP24 (Fig. 1f). These data mirror the results from the immunoprecipitations, and they further support the conclusion that NP, VP35, and VP24 interact with each other in the absence of any other viral protein.

### VP24 amino acids V170 and N171 are critical for interaction with NP

**Figure 2:**
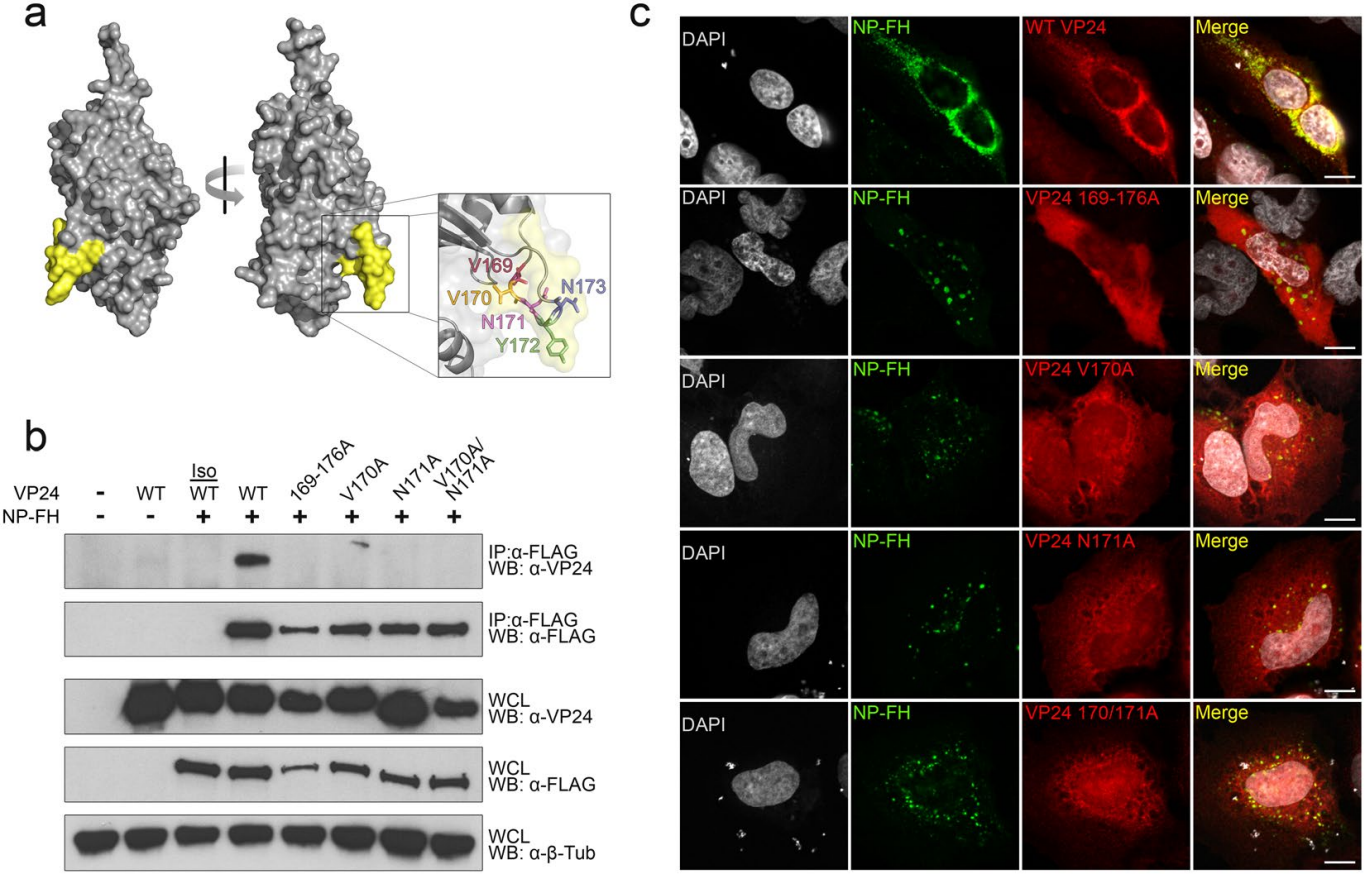
VP24 amino acids V170 and N171 are critical for interacting with NP. (**a**) A structural model of EBOV VP24 based on the crystal structure of Sudan and Reston virus VP24 (PDB 3VNE & 4D9O). Amino acids 169–176 are colored yellow on the surface diagram, and the inset highlights amino acids 169–173 with a ribbon and stick diagram. The arrow indicates a 90° rotation of the molecule around the Y-axis. (**b**) HEK 293 cells were co-transfected with pCAGGS-NP-FH and pCAGGS-Wild-type (WT) VP24 or a VP24 point mutant: pCAGGS-VP24 169–176A, pCAGGS-VP24 V170A, pCAGGS-VP24 N171A, or pCAGGS-VP24 V170A/N171A. Lysates were immunoprecipitated with mouse anti-FLAG or isotype control (Iso) antibodies, and immunoprecipitation (IP) and whole cell lysate (WCL) fractions were subjected to Western blot (WB) with mouse anti-FLAG, rabbit anti-VP24, or rabbit anti-β-tubulin antibodies. IP/Western blot data are representative of at least three independent experiments. Western blots were cropped for presentation; full-length blots are available in Supplementary Fig. 7. (**c**) Hela cells were co-transfected with pCAGGS-NP-FH and pCAGGS-Wild-type (WT) VP24 or a VP24 mutant: pCAGGS-VP24 169–176A, pCAGGS-VP24 V170A, pCAGGS-VP24 N171A, or pCAGGS-VP24 V170A/N171A. Following fixation and permeabilization, cells were stained with mouse anti-FLAG and rabbit anti-VP24 antibodies prior to staining with AlexaFluor 488 goat anti-mouse and AlexaFluor 546 goat anti-rabbit secondary antibodies. Coverslips were mounted with ProLong Gold Antifade Mountant containing DAPI to visualize the nuclei. Note that the extranuclear DAPI staining is an occasional artifact of transfection. White scale bars represent 10 uM.

To determine the region of VP24 that was responsible for interacting with NP, we developed a structural model of EBOV VP24 based on the crystal structures of VP24 from the related Sudan and Reston viruses (Fig. 2a)^21^. We then used four protein-protein interaction prediction algorithms to predict regions on our EBOV VP24 structural model that would be most likely to interact with other proteins, the consensus of which is depicted in Supplementary Fig. 2a. Notably, this prediction strongly suggested the involvement of amino acids 169–180 in protein-protein interaction (Supplementary Fig. 2a). We next generated a series of VP24 mutants with the putative interaction regions mutated to alanines (Supplementary Fig. 2a), and subsequently used co-immunoprecipitation to assess their ability to interact with NP (Supplementary Fig. 2b). Whereas the majority of the mutants retained their ability to interact with NP, VP24 mutant 169–176A neither interacted, nor co-localized with, NP, suggesting that these eight amino acids were in fact important for the ability of VP24 to interact with NP (Fig. 2b,c and Supplementary Fig. 2b). Since mutating amino acids 174–180 did not abrogate interaction with NP (Supplementary Fig. 2b), we reasoned that amino acids 169–173, which are highly conserved among all filoviruses (Supplementary Fig. 3a), were most critical. Individual mutation of each of these five amino acids to alanines revealed that V170 and N171 were both necessary for the ability of VP24 to interact with NP (Supplementary Fig. 3b). Mutation of either or both V170 and N171 to alanines abolished the interaction between VP24 and NP (Fig. 2b), and all of these mutations exhibited reduced co-localization with NP (Fig. 2c). Notably, we did observe a change in NP localization when interaction between NP and VP24 was lost, and we speculate that these NP puncta may represent aggregates of malformed nucleocapsids, although further investigation is required (Fig. 2c). Thus, using a structural bioinformatics approach, we predicted a region on VP24 composed of amino acids 169–173 that we experimentally confirmed is critical to the ability of VP24 to interact with NP, and we resolved this interaction domain to the amino acid level, identifying V170 and N171 as two amino acids required for interaction with NP.

### VP24 mutants do not support the EBOV replication cycle

**Figure 3:**
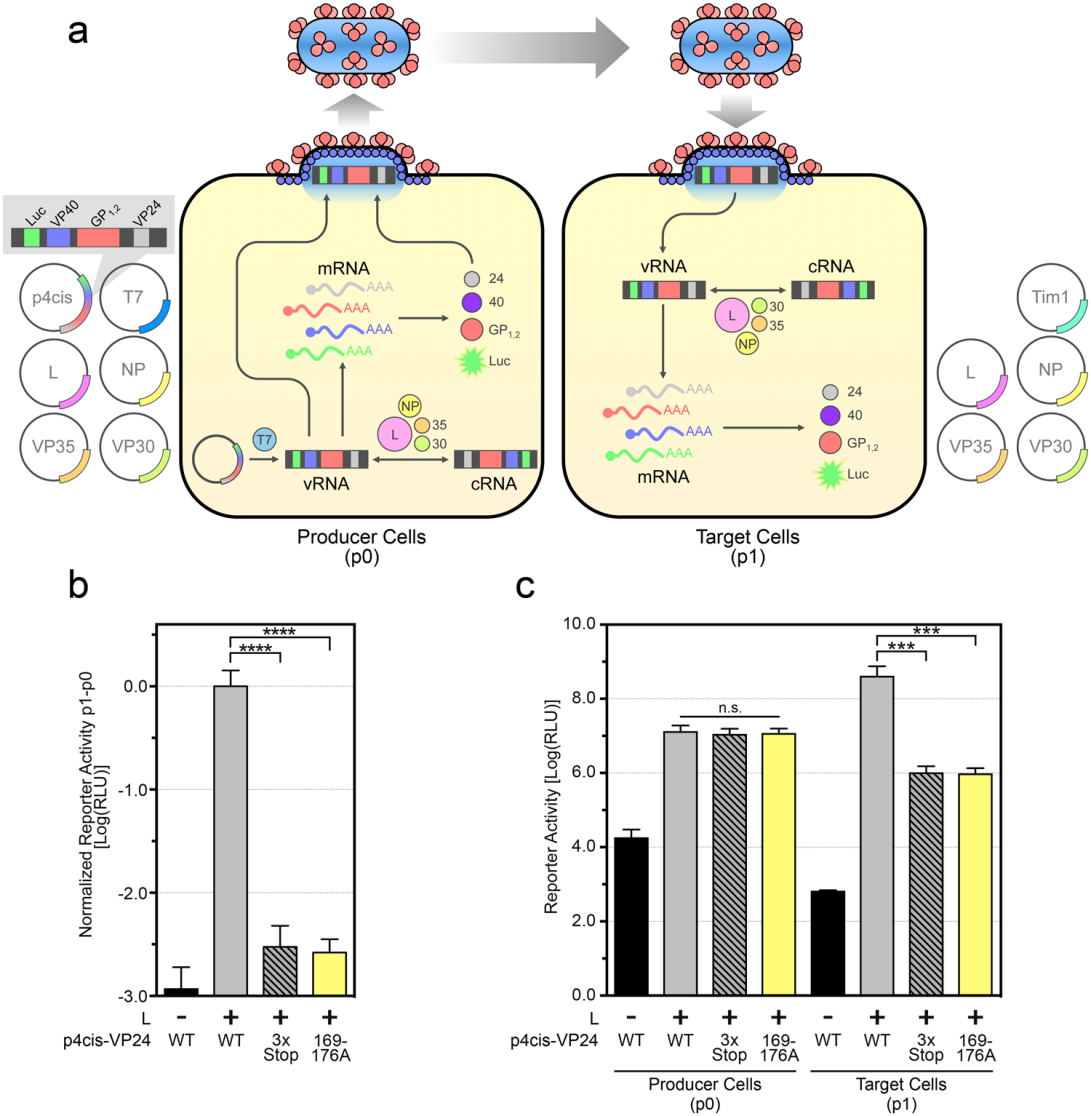
VP24 169–176A does not support trVLP production. (**a**) A schematic of the EBOV transcription/replication-competent virus-like particle (trVLP) assay. In producer cells (p0), initial transcription of the tetracistronic minigenome (4xMG) by the T7 polymerase produces a viral RNA (vRNA) template, which is then replicated, via complementary RNA (cRNA) intermediates, by the EBOV ribonucleoprotein complex, consisting of L, NP, VP35, and VP30. From the minigenome, the ribonucleoprotein complex also transcribes Renilla luciferase, VP40, GP_1,2_, and VP24 mRNA, which is then translated by host cellular machinery. NP encapsidates the vRNA, along with VP35, VP30, VP24, and L, and the nucleocapsids interact with the matrix protein VP40 and the glycoprotein GP_1,2_, to form trVLPs. trVLPs are capable of infecting target cells (p1) pre-transfected with the EBOV ribonucleoprotein complex and mounting a second round of replication. Renilla luciferase signal in producer cells is proportional to EBOV minigenome replication and transcription, and in target cells it is additionally proportional to the amount of trVLPs produced and capable of infecting target cells. Notably, VP24 is required for the production of infectious trVLPs. (**b**,**c**) HEK 293T producer cells were co-transfected with p4cis-vRNA-RLuc (p4cis), encoding wild-type (WT) VP24, VP24-3x-stop, or VP24 169–176A, as well as pCAGGS-luc2, encoding Firefly luciferase as a transfection control, pCAGGS-T7, pCAGGS-L, pCAGGS-NP, pCAGGS-VP35, and pCAGGS-VP30. trVLPs in the supernatant were harvested and used to infect HEK 293T target cells pre-transfected with pCAGGS-Tim1, pCAGGS-L, pCAGGS-NP, pCAGGS-VP35, and pCAGGS-VP30. Data are presented as relative light units (RLU) on a log scale with the Renilla luciferase signal in target cells (p1) minus the Renilla luciferase signal in producer cells (p0) normalized to WT VP24 (**b**) or with the Renilla luciferase signals in producer (p0) and target cells (p1) indicated separately (**c**). The Renilla luciferase signal in producer cells was normalized against the average Firefly luciferase signal. The means and standard error of the mean for 3 independent experiments are shown (n.s., not significant; ***p ≤ 0.001; ****p ≤ 0.0001).

To better understand the function of our VP24 mutants—and, by extension, the VP24-NP interaction—we next sought to characterize the ability of these mutants to support the EBOV replication cycle. To this end we employed the EBOV tetracistronic transcription/replication-competent virus-like particle (trVLP) system (Fig. 3a)^22^. Producer cells (also known as “p0”) were transfected with the components required to produce trVLPs, including a tetracistronic minigenome expressing wild-type VP24 (p4cis-VP24 WT), a tetracistronic minigenome incapable of expressing WT VP24 due to three stop codons placed immediately after the VP24 start codon (p4cis-VP24-3x-stop), or a tetracistronic minigenome expressing VP24 169–176A (p4cis-VP24 169–176A). Seventy-two hours post-transfection, cell supernatants were transferred onto pre-transfected target cells (also known as “p1”) and producer cell lysates were harvested. After an additional 72 hours, target cells were lysed and Renilla luciferase activity was measured in both producer and target cells. Results are expressed as luciferase activity in target cells (p1) minus that in producer cells (p0), normalized to WT VP24 (Fig. 3b). In the absence of the polymerase L, minigenome replication and transcription is impossible, thereby preventing trVLP production in producer cells and eliminating luciferase reporter activity in target cells. Similarly, in the absence of WT VP24, significantly less luciferase activity was detected in target cells compared to cells inoculated with WT trVLPs, indicating a defect in the production or infectivity of trVLPs produced in the absence of VP24. Intriguingly, VP24 169–176A also did not support the production of functional trVLPs. The same data, presented as individual producer (p0) and target (p1) cell luciferase activity, demonstrated that the absence of VP24 or the presence of VP24 169–176A did not affect replication and transcription in producer cells, implying a defect in trVLP production downstream of these processes (Fig. 3c). Together these data demonstrate that the VP24-NP interaction is absolutely critical for the completion of the EBOV replication cycle, not by facilitating replication/transcription but instead, we hypothesize, by facilitating the structural formation of the trVLPs themselves.

### VLPs produced with mutant VP24 contain reduced levels of EBOV minigenome RNA

**Figure 4:**
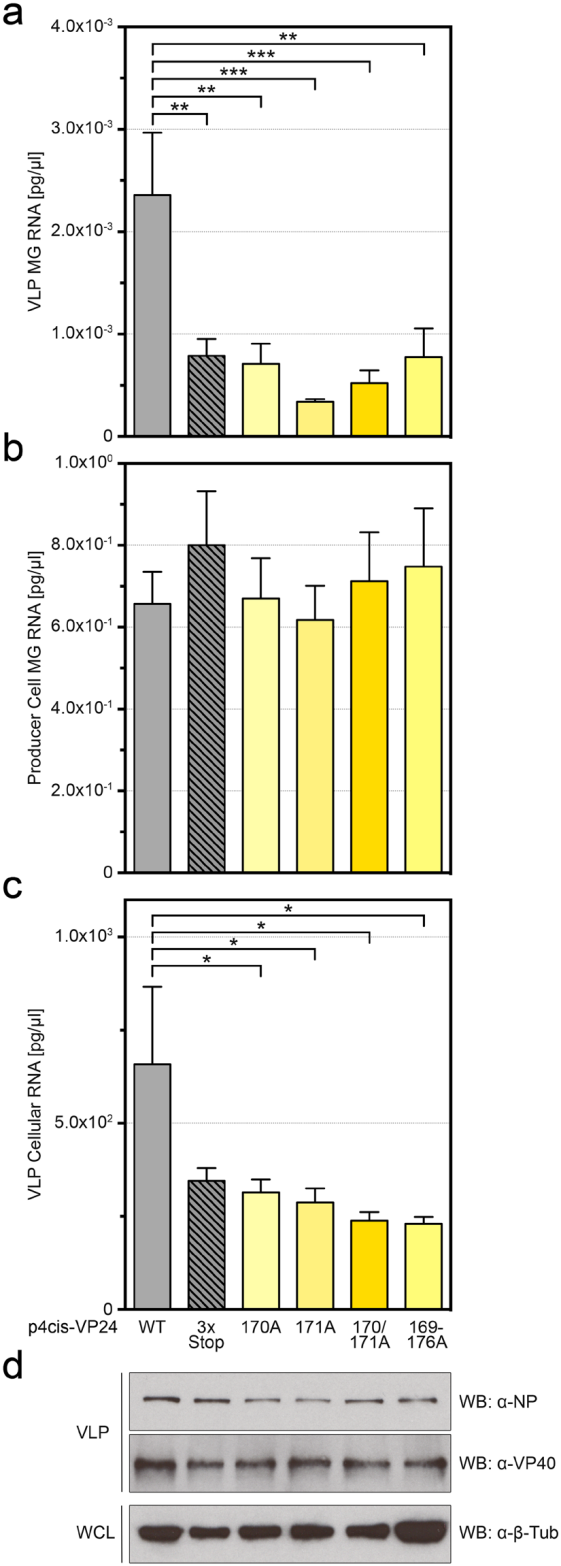
VLPs produced with mutant VP24 contain reduced levels of EBOV minigenome. (**a**–**d**) HEK 293 cells were co-transfected with p4cis-vRNA-RLuc, encoding wild-type (WT) VP24, VP24-3x-stop, VP24 V170A, VP24 N171A, VP24 V170A/N171A or VP24 169–176A, as well as pCAGGS-T7, pCAGGS-L, pCAGGS-NP, pCAGGS-VP35, and pCAGGS-VP30. Supernatant was concentrated and virus-like particles (VLPs) were purified over a 20% sucrose cushion via ultracentrifugation. Following lysis of purified VLPs, viral minigenome RNA was purified and quantified via RT-qPCR (**a**) or total cellular RNA was quantified using a Low Abundance RNA Quantification kit (**c**). Producer cells were lysed, and viral minigenome RNA was purified and quantified via RT-qPCR (**b**). Data are presented as pg/ul EBOV minigenome RNA isolated from VLPs (**a**) or producer cells (**b**) or as pg/ul total cellular RNA isolated from VLPs (**c**). The means and standard error of the mean for 5 independent experiments are shown (*p ≤ 0.05; **p ≤ 0.01; ***p ≤ 0.001). VLP lysates and producer cell whole cell lysates (WCL) were subjected to Western blot (WB) with mouse anti-NP, rabbit anti-VP40, and rabbit anti-β-tubulin antibodies (**d**). Western blots were cropped for presentation; full-length blots are available in Supplementary Fig. 8.

The inability of VP24 169–176A to support the production of trVLPs can be explained either by the lack of efficient nucleocapsid packaging into the virion or by a defect downstream of trVLP formation, such as infection of target cells or release of nucleocapsid into target cells. To address these possibilities, we sought to quantify the amount of minigenome RNA packaged into VLPs. Similarly to the previous assay (Fig. 3a), cells were transfected with plasmids encoding NP, VP35, VP30, and L along with the T7 polymerase and the tetracistronic minigenome encoding WT or mutant VP24. Seventy-two hours post-transfection, supernatants were harvested and concentrated, followed by purification of VLPs over a sucrose cushion. Purified VLPs were lysed and their EBOV minigenome RNA content was quantified by RT-qPCR. Compared to trVLPs generated with WT VP24, VLPs generated with no VP24 or mutant VP24, including VP24 169–176A, V170A, N171A, and V170A/N171A, contained significantly less EBOV minigenome RNA (Fig. 4a), despite the fact that similar concentrations of total minigenome RNA were present in producer cells (Fig. 4b). Intriguingly, the concentration of total cellular RNA isolated from trVLPs generated with WT VP24 was also higher than that isolated from VLPs generated with no or mutant VP24 (Fig. 4c), suggesting that illegitimate encapsidation and packaging of non-viral RNA was also much more efficient in the presence of WT VP24. Moreover, VLPs generated with no or mutant VP24 appeared to contain less NP in the absence of VP24 or the presence of mutant VP24, indicating a decrease in nucleocapsid packaging (Fig. 4d and Supplementary Fig. 4). Interestingly, no consistent difference in VP40 levels was observed, suggesting that virion budding, unlike nucleocapsid packaging, was unaffected (Fig. 4d and Supplementary Fig. 4). Together, these data suggest that VP24 plays a key role in nucleocapsid formation and they imply that, in the absence of efficient nucleocapsid formation, packaging of nucleocapsids into virions is impaired.

### VP24 facilitates genome encapsidation

**Figure 5:**
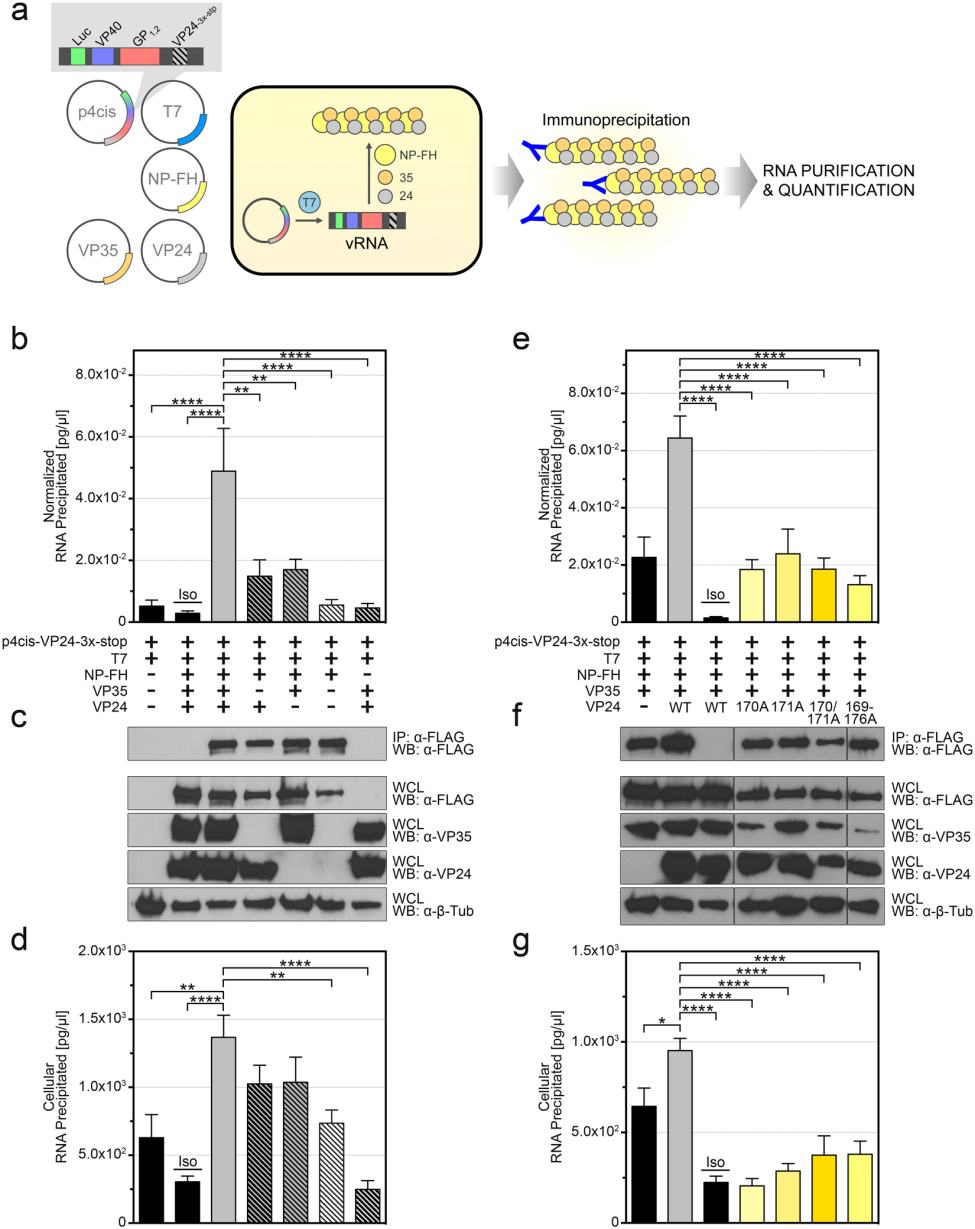
VP24 is critical for EBOV genome encapsidation. (**a**) A schematic of the EBOV RNA immunoprecipitation assay. Transcription of the tetracistronic minigenome (p4cis), encoding VP24-3x-stop, by the T7 polymerase produces viral RNA (vRNA) template that can be encapsidated by NP with a C-terminal FLAG/HA tag (NP-FH), VP35, and VP24, supplied *in trans*. (**b**–**g**) HEK 293 cells were co-transfected with p4cis-vRNA-RLuc (p4cis), encoding VP24-3x-stop, as well as pCAGGS-T7, pCAGGS-NP-FH, pCAGGS-VP35, and wild-type (WT) pCAGGS-VP24 (**b**–**d**) or a VP24 mutant: pCAGGS-VP24 V170A, pCAGGS-VP24 N171A, pCAGGS-VP24 V170/N171A, or pCAGGS-VP24 169–176A (**e**–**g**). Lysates were immunoprecipitated with mouse anti-FLAG or an isotype control (Iso) antibody, and RNA was purified and then quantified via RT-qPCR. The total concentration in pg/ul of EBOV minigenome RNA precipitated was normalized against the total concentration of EBOV minigenome RNA in whole cell lysates for each transfection (**b**,**e**). Immunoprecipitation (IP) and whole cell lysate (WCL) fractions were subjected to Western Blot (WB) with mouse anti-FLAG, mouse anti-VP35, rabbit anti-VP24, or rabbit anti-β-tubulin antibodies (**c**,**f**). Western blots were cropped for presentation; full-length blots are available in Supplementary Fig. 9. The total concentration in pg/ul of cellular RNA in IP fractions was quantified using a Low Abundance RNA Quantification kit (**d**,**g**). The means and standard error of the mean for 8 (**b**) and 6 (**e**) independent experiments are shown, with the exception of the minigenome/T7-only control, for which 7 (**b**) independent experiments are shown (**p ≤ 0.01; ****p ≤ 0.0001).

Evidence suggests that VP24 is responsible for altering the structure of the viral nucleocapsid, condensing it into a conformation that is more efficiently packaged into virions^13, 14, 16, 20, 22–27^. Indeed, it has been postulated that this structural condensation serves as a switch that moves the viral replication cycle from the replication/transcription phase to the packaging and egress phase^20, 22, 28^. This would explain the ability of excess WT VP24, but not VP24 169–176A, to inhibit minigenome activity (Supplementary Fig. 5), and it is consistent with the loss of minigenome RNA in VLPs produced in the absence of VP24 or the presence of a VP24 mutant (Fig. 4). In an effort to more directly assess the ability of VP24 to condense the nucleocapsid, we developed an RNA immunoprecipitation assay to quantify the amount of EBOV minigenome RNA that interacts with the EBOV nucleocapsid (Fig. 5a). Cells were transfected with plasmids encoding the nucleocapsid components NP-FH, VP35, and VP24, as well as p4cis-VP24-3x-stop and a plasmid encoding the T7 polymerase to provide a source of viral RNA. Seventy-two hours post-transfection, cells were lysed and subjected to immunoprecipitation with an anti-FLAG antibody, followed by RNA extraction and quantification by RT-qPCR. Only in the presence of all three EBOV nucleocapsid components was RNA precipitation successful (Fig. 5b). In the absence of either or both VP35 and VP24, RNA precipitation was significantly reduced, as was the case when only NP-FH was absent or when an isotype control antibody was used (Fig. 5b). Western blotting of lysates demonstrated that NP was evenly precipitated and that all proteins were expressed to similar levels (Fig. 5c). Overall, these data demonstrate that VP24, along with NP and VP35, are critical for the efficient precipitation of EBOV minigenome RNA, presumably by facilitating its encapsidation and condensation. Notably, a higher concentration of cellular RNA was detected in IP samples when all three nucleocapsid components were present, although the difference was only statistically significant when both VP35 and VP24 were absent or when NP was absent (Fig. 5d). Nevertheless, these data also suggest that both viral and cellular RNA are encapsidated and condensed by NP, VP24, and VP35, and they imply that both VP24 and VP35 are critical for this process. Analysis of the VP24 mutants revealed that VP24 169–176A, as well as the point mutants V170A, N171A, and V170A/N171A, all exhibited significantly less precipitated EBOV RNA than was observed with WT VP24 (Fig. 5e), indicating that the interaction between VP24 and NP was critical for the encapsidation of the EBOV minigenome. Western blotting of lysates demonstrated that NP was evenly precipitated in this case and that all proteins were expressed to similar levels, although we did observe a reduction in VP35 expression when co-expressed with VP24 169–176A (Fig. 5f). We once again observed a higher concentration of total cellular RNA in the IP samples when WT VP24 was present—and in this case the differences were all statistically significant—indicating that the VP24 mutants unable to interact with NP were also unable to promote efficient RNA encapsidation (Fig. 5g). Overall, these data demonstrate a direct role for VP24 in EBOV nucleocapsid formation and along with the data in Fig. 4 they suggest that the lack of trVLP production observed in the absence of VP24 or the presence of mutant VP24 (Fig. 3) is likely a result of inefficient nucleocapsid formation leading to inefficient packaging of nucleocapsids into virions.

## Discussion

Although VP24 is occasionally referred to as the minor matrix protein of EBOV^1^, mounting evidence suggests that it instead plays a central role in nucleocapsid formation, along with NP and VP35^13, 14, 16, 23, 26^. Nevertheless, the molecular details of VP24’s contribution to nucleocapsid formation have, until now, remained largely unexplored. Here we present data demonstrating a direct role for VP24 in genome packaging and nucleocapsid condensation—a function that is dependent on VP24’s ability to interact with NP. Through its interaction with NP, which was dependent on VP24 amino acids V170 and N171, VP24 facilitated the condensation of the viral nucleocapsid, thereby ensuring its efficient packaging and the production of infectious virions. This study highlights the critical role that VP24 plays in nucleocapsid assembly and genome packaging, and it identifies new targets for the development of antiviral therapeutics.

Although previous studies have demonstrated an interaction between NP and VP24 that appears weak or transient^14, 20, 29^, we were able to detect a robust and consistent interaction between the two wild-type proteins by immunoprecipitation (Figs 1 and 2 and Supplementary Figs 1–3). Such an interaction might be predicted given the integral role VP24 plays in nucleocapsid structure, and it is consistent with cryo-electron microscopy modeling that indicates VP24 is a significant part of the nucleocapsid, with the molar ratio of VP24 to NP estimated to be at least 1:1^12, 23^. Moreover, a series of alanine scanning mutants allowed us to identify amino acids 169–173 as being important to the ability of VP24 to interact with NP, and further analyses identified V170 and N171 as critical amino acids within this region. The NP-binding region we identified was predicted to be on an exposed loop in our model of VP24, and the loop structure and accessibility was subsequently confirmed by the published crystal structure^30^. This loop is highly conserved among filoviruses: the entire eight-amino acid region is 100% identical between EBOV and the related viruses Reston, Tai Forest, and Bundibugyo, while Sudan virus differs by only one amino acid, N173K (Supplementary Fig. 3a). While the more distantly related Marburg virus is more divergent in this region, like the other filoviruses, it still possesses the critical amino acids V170 and N171. Lloviu virus also possesses N171 but lacks the valine at position 170, which is instead replaced by the highly similar isoleucine. The conservation observed in this region of VP24 underscores the importance of these amino acids to the interaction with NP, and it suggests that all filoviruses rely upon these amino acids to mediate such an interaction. Notably, although we identified other VP24 mutants that exhibited a reduced or absent interaction with NP, these constructs were relatively poorly expressed, making their interaction profile difficult to conclude (Supplementary Fig. 2b). VP24 N194A, which appeared to have lost its interaction with NP, was especially interesting since the N194A mutation is proximal to amino acids 169–173 in our structural model; however, over-expression of this mutant revealed a robust interaction with NP that was not present when the protein was poorly expressed, as in Supplementary Fig. 2b (data not shown). Nevertheless, we cannot rule out the possibility that other regions of VP24 also play a role in interacting with NP.

Numerous studies over the last several years have identified a role for VP24 in the structural formation of the nucleocapsid, along with NP and VP35^13–15^. In particular, VP24 has been hypothesized to condense the nucleocapsid into a structure that is sufficient for packaging into the nascent virion^22^, and the absence of VP24, or the inability of VP24 to interact with NP, would be expected to disrupt the formation of a condensed nucleocapsid. Using an RNA IP approach, we were able to precipitate EBOV nucleocapsids and detect viral RNA, but only in the presence of all three nucleocapsid components (Fig. 5). In the absence of VP24 or in the absence of an interaction between VP24 and NP, we were unable to precipitate RNA, presumably because the unstructured and loosely coiled nucleocapsid was less efficiently precipitated and/or unable to protect the minigenome throughout the precipitation protocol. These uncondensed nucleocapsids were also less efficiently packaged into virions, as demonstrated by the significantly reduced viral minigenome RNA content of purified trVLPs (Fig. 4), which, in turn, explains the inability of VP24 169–176A to facilitate the production of trVLPs (Fig. 3). Moreover, the inability of VP24 169–176A to interact with NP and promote the formation of condensed nucleocapsids likely explains the inability of this mutant to inhibit minigenome replication/transcription. VP24’s ability to inhibit viral replication and transcription has been previously described and assumed to be a direct consequence of VP24 interacting with NP to condense the nucleocapsid and restrict the EBOV polymerase complex from accessing and/or moving along the genome^20, 28^. In this way, VP24, which is expressed in relatively low abundance later during the viral replication cycle^31, 32^ is thought to act as a kind of “molecular switch” that turns off replication and transcription and therefore permits packaging and egress. Accordingly, in the absence of any interaction with NP, VP24 was unable to condense the nucleocapsid and therefore unable to inhibit minigenome replication/transcription (Supplementary Fig. 5). Overall, these data demonstrate the critical role played by the VP24-NP interaction in nucleocapsid formation, and they offer compelling evidence of VP24’s ability to promote nucleocapsid condensation.

Although there exists a considerable amount of evidence in support of the theory that VP24 is required for EBOV nucleocapsid condensation, there is intriguing evidence to the contrary as well. As assessed by cryo-electron microscopy, Bharat and colleagues demonstrated that the interaction between VP40 and NP is sufficient to condense the nucleocapsid and that the addition of VP35 and VP24 serves only to further rigidify the structure^16^. Nevertheless, it is clear from numerous other electron microscopy studies that VP24, VP35, and NP, alone, form nucleocapsids that are indistinguishable from those produced during EBOV infection^13–16^. How can these disparate observations be reconciled? In the context of virus infection, individual EBOV nucleocapsids have been observed to leave perinuclear inclusion bodies and be transported in an actin-dependent manner as discrete units to the site of virion budding at the plasma membrane^33^. We hypothesize that transport of an uncondensed, loosely coiled nucleocapsid is highly inefficient, if not impossible, making nucleocapsid condensation a prerequisite for packaging and ensuring that nucleocapsids rarely, if ever, encounter VP40 at the plasma membrane prior to condensation. Our data are consistent with this interpretation, demonstrating that VP24 is required for the condensation of the nucleocapsid (Fig. 5) and its subsequent packaging into the virion (Fig. 4). Indeed, we observed less NP packaged into VLPs when VP24 was absent or mutated (Fig. 4d), and this is in accordance with data obtained using siRNA knockdown of VP24 during virus infection^24^. These data are also consistent with the observation that VP24 operates in a species-specific manner: in the context of recombinant EBOV infection, WT VP24, but not guinea pig-adapted VP24, produces massive protein aggregates in guinea pig macrophages, instead of the characteristic inclusion bodies containing nucleocapsids^25^. Presumably these aggregates are made up of malformed nucleocapsids that are not suitable for transport to the plasma membrane. It is interesting to speculate that malformed nucleocapsid aggregation may also be responsible for the change in NP localization from cytoplasmic to punctate that we observed via immunofluorescence microscopy when VP24 lost its ability to interact with NP (Fig. 2c). Regardless, while transient over-expression of VP40 may “condense” nucleocapsids in the context of concomitant NP over-expression, this scenario may not accurately reflect EBOV infection.

It is worth noting that we detected significant amounts of illegitimate encapsidation/condensation of cellular RNA when NP, VP35, and WT VP24 were expressed (Fig. 5d,g), and this correlated with the increased detection of cellular RNA in purified trVLPs produced with WT VP24 (Fig. 4c). Since NP alone is capable of binding to and oligomerizing around single-stranded RNA from any source, including viral, cellular, and bacterial^16, 17, 34^, it was not surprising to find encapsidated cellular RNA. Indeed, this observation further underscores the contribution of VP24 to the structural formation of the nucleocapsid and its subsequent packaging. However, whether this observation is relevant to virus infection is unclear; to our knowledge the total RNA content of EBOV virions has yet to be thoroughly defined and quantified.

Notably, VP35 was also required for the encapsidation of viral RNA (Fig. 5b), a result that is not necessarily surprising given that VP35 is an integral component of the EBOV nucleocapsid^13–15^. Indeed, we demonstrated independent interactions between VP35 and NP and VP35 and VP24 (Fig. 1). Although the NP-VP35 interaction has been known for some time^18, 35, 36^, the interaction between VP35 and VP24 had until now never been demonstrated, despite attempts^20^. The reasons for this are unclear since the interaction was not only robust and consistent in our hands but also predicted based on cryo-electron microscopy studies that suggest VP35 and VP24 form a bridge linking NP monomers within the nucleocapsid^23^. Interestingly, VP35 has been suggested to function as a chaperone for NP, guiding NP monomers to newly synthesized viral RNA in a manner analogous to the phosphoprotein and nucleoprotein of vesicular stomatitis virus, a related negative-sense RNA virus^35–37^. Whether VP24 contributes to or otherwise affects the chaperone activity of VP35 is unknown, but considering the interactions between VP24, VP35, and NP, it is conceivable. Indeed, our data are consistent with a role for VP24 in selectively encapsidating viral (versus cellular) RNA (Figs 4 and 5). Future work will focus on the interaction between VP35 and VP24, as well as between NP and VP35, in an effort to understand in more detail the functional relationships between these proteins.

Interactions among viral proteins highlight important potential targets for drug development. Not only have we identified the molecular determinants of the interaction between VP24 and NP, but we have also shown this interaction to be structurally and functionally critical to the EBOV replication cycle. As such, the interface between VP24 and NP represents an exciting and convincing target that, if disrupted, could have specific and significant anti-viral effects, possibly reducing off-target effects that might plague compounds identified by high-throughput screening. Indeed, VP24 has been identified as a key virulence factor for EBOV that, when disrupted, confers protection against lethal EBOV infection^38^. Furthermore, since the interaction between VP24 and NP is presumably highly conserved, a drug that targets the interaction interface may be effective against filoviruses other than EBOV. Given the recent and devastatingly large outbreak of EBOV in Western Africa, and the potential for other filoviruses to cause similar outbreaks, highly specific and effective anti-filoviral countermeasures are urgently needed. This work provides the basis for identifying novel therapeutics that specifically target the critical interaction between NP and VP24.

## Methods

### Cells & Viruses

HeLa, human embryonic kidney (HEK) 293, HEK 293T, and Vero E6 cells were obtained from the ATCC and maintained in Dulbecco’s Modified Eagle’s medium (Sigma Aldrich), supplemented with 10% heat-inactivated fetal bovine serum (HI-FBS; Thermo Fisher), 2mM L-glutamine (Thermo Fisher), 50 U/ml penicillin, and 50 ug/ml streptomycin (Thermo Fisher) at 37 °C and 5% CO_2_. Huh7 cells were kindly provided by Dr. Yoshiharu Matsuura (Osaka University) and were maintained as above.

Rescue of recombinant EBOV strain Mayinga encoding NP with an N-terminal FLAG/HA tag [EBOV (NP-FH)] from full-length cDNA was performed using a procedure described previously^39^. EBOV (NP-FH), along with WT EBOV strain Mayinga, was propagated and titrated on Vero E6 cells and stored in liquid nitrogen. All work with infectious Ebola viruses was performed in the biosafety level 4 laboratories of the Integrated Research Facility at Rocky Mountain Laboratories, Division of Intramural Research, National Institute of Allergy and Infectious Diseases, National Institutes of Health in accordance with standard operating protocols approved by the Rocky Mountain Laboratories Institutional Biosafety Committee.

### Plasmids & Cloning Methodology

EBOV expression plasmids pCAGGS-L, pCAGGS-NP, pCAGGS-VP30, and pCAGGS-VP35 were generated as previously described^39^. All other EBOV protein expression plasmids were generated by PCR amplification of the open-reading frames (ORFs) of interest from sub-genomic plasmids, as described elsewhere^39^, followed by insertion into the expression plasmid pCAGGS using standard cloning techniques. A Kozak consensus sequence was placed immediately upstream of the start codon of all EBOV ORFs. The monocistronic minigenome plasmid (p1cis-vRNA-RLuc), encoding Renilla luciferase, as well as the tetracistronic minigenome plasmids encoding Renilla Luciferase, VP40, GP_1,2_ and either the open reading frame (ORF) for wild type VP24 (p4cis-vRNA-RLuc) or a VP24 ORF possessing three stop codons immediately after the start codon (p4cis-vRNA-RLuc-VP24-3x-stop) were generated as previously described^22, 27^. Restriction digestion was used to subclone mutant VP24 sequences from pBS-EBOV-4728-11860 into the EBOV minigenome subcloning vector pKan1.5-MscI-XmaI, from which restriction digestion was used to clone the plasmid p4cis-vRNA-RLuc-VP24 169–176A. To generate p4cis-vRNA-RLuc-VP24 V170A, N171A, and V170A/N171A, point mutations were generated in pKan1.5-MscI-XmaI, followed by restriction digestion and insertion into p4cis-vRNA-RLuc. pCAGGS-TIM-1 and pCAGGS-T7 were generated as previously described^22, 27^. pCAGGS-T7(K179C/M750C) was generated with the point mutations K179C and M750C to help reduce early termination events^40^. The sequences of all plasmids generated in this study were confirmed by Sanger sequencing.

### Protein Immunoprecipitation

HEK 293 cells (1 × 10^6^ cells) were transfected with the indicated pCAGGS-based plasmids using 3 ul of the transfection reagent TransIT-LT1 (Mirus) per 1 ug DNA according to manufacturer’s instructions. The total amount of DNA for each transfection was kept constant in each experiment by complementing with empty-vector pCAGGS. Cells were lysed in 1% Igepal lysis buffer [150 mM NaCl, 50 mM Tris pH 7.4, 1% Igepal CA-630 (Sigma) supplemented with cOmplete EDTA-free protease inhibitor cocktail tablets (Roche)], followed by immunoprecipitation with mouse anti-FLAG M2 antibody (Sigma), mouse anti-c-Myc 9E10 antibody (Sigma), or mouse isotype control antibody MOPC21 (Sigma). Immune complexes were precipitated with Dynabeads conjugated to protein G (Thermo Fisher) and re-suspended in 2x Laemmli sample buffer (Bio-Rad). A portion of pre-immune whole cell lysate was reserved and subjected to acetone precipitation; precipitated protein was re-suspended in 2x Laemmli sample buffer. Similar experiments were performed in the context of EBOV infection: VeroE6 cells (1 × 10^7^ cells) were infected with WT EBOV or EBOV (NP-FH) at an MOI of 1, and four days post-infection, cells were lysed in 1% NP-40 lysis buffer, followed by immunoprecipitation with mouse anti-FLAG M2 antibody or mouse isotype control antibody MOPC21. Immune complexes were precipitated with Dynabeads conjugated to protein G and re-suspended in 4x Laemmli sample buffer [200 mM Tris pH 6.8, 4% sodium dodecyl sulfate (SDS), 35% glycerol, 0.05% bromophenol blue, 20% beta-mercaptoethanol]. A portion of pre-immune whole cell lysate was reserved and mixed with 4x Laemmli sample buffer. All EBOV sample inactivation and removal from the BSL-4 laboratory was performed according to standard operation protocols approved by the local institutional biosafety committee.

### Immunoblotting and Antibodies

Cell lysates were subjected to SDS polyacrylamide gel electrophoresis (SDS-PAGE) and proteins were transferred onto polyvinylidene fluoride or nitrocellulose membranes. The following primary antibodies were used for detection of proteins by Western blot: mouse anti-FLAG M2 (1:1000), mouse anti-c-Myc 9E10 (1:5000), rabbit anti-beta-tubulin (1:1000; Abcam), rabbit anti-VP24 (1:2000), mouse anti-NP 74-7 (1:1000; a kind gift from Dr. Ayato Takada, Hokkaido University^41^), rabbit-anti-VP40^39^ (1:1000), and mouse anti-VP35 (1:1000; a kind gift from Dr. Christopher Basler, Georgia State University). Donkey anti-mouse and donkey anti-rabbit secondary antibodies conjugated to horseradish peroxidase (1:50000; Jackson ImmunoResearch Laboratories) were used along with enhanced chemiluminescence substrate (Promega) to visualize protein signal.

To produce rabbit anti-VP24, a New Zealand White rabbit was immunized intramuscularly with TiterMax Gold Adjuvant (Sigma) plus 350 ug of purified VP24 peptide corresponding to amino acids 5–20 and conjugated to keyhole limpet hemocyanin at the N-terminus (Bio-Synthesis). Following the initial immunization, the rabbit was boosted two more times with 350 ug VP24 peptide in Freund’s Incomplete Adjuvant (Sigma), followed by a fourth and final immunization with 175 ug VP24 peptide in Freund’s Incomplete Adjuvant. Immunizations were administered approximately every two weeks, and two weeks after the final immunization, the rabbit was euthanized and blood samples were obtained, from which serum was separated. Animal studies were approved by the institutional animal care and use committee and were performed by certified staff in facilities approved by the Association for Assessment and Accreditation of Laboratory Animal Care.

### Confocal Microscopy

HeLa cells (~4 × 10^4^ cells) were grown on 8-well Nunc Lab-Tek II Chamber Slides (Thermo Fisher) and transfected with the indicated plasmids using TransIT-LT1 according to manufacturer’s directions. Following fixation in 10% formalin, cells were permeabilized in 0.25% Triton X-100 (Sigma), and blocked in 30% goat serum (Sigma). The primary antibodies mouse anti-FLAG M2 (1:200), rabbit anti-FLAG (1:200; Sigma), mouse anti-c-Myc 9E10 (1:200; Santa Cruz), and rabbit anti-VP24 (1:200) were used along with the secondary antibodies AlexaFluor 488 goat anti-mouse and AlexaFluor 546 goat anti-rabbit (1:400; Thermo Fisher). Coverslips were mounted using ProLong Gold Antifade Mountant with DAPI (Thermo Fisher), and cells were examined using a Zeiss LSM710 confocal microscope.

### Structural Bioinformatics Analyses

A theoretical model for amino acids residues 9–231 of EBOV VP24 (GI: 21702654) was built with Rosetta3^42^ using the Sudan and Reston virus VP24 crystal structures^21^ (PDB 3VNE & 4D9O) as templates. The sequences of EBOV, Sudan, and Reston VP24 were aligned, and the crystal structures of Sudan and Reston virus VP24 were superposed. Based on the C-alpha root mean square deviations (RMSD), location of amino acid identities, and insertions or deletions relative to EBOV VP24, a hybrid template was produced from the Sudan and Reston crystal structures. Using Rosetta3, the bond lengths and angles of the hybrid template were idealized, after which the sequence of EBOV VP24 was designed onto the template using the RosettaDesign algorithm^43^, and the backbone conformation and rotamers of the designed model were minimized together in the Rosetta energy function using the FastRelax algorithm^44^. Potential protein-protein interaction motifs were identified by analyzing the theoretical model using the following computational methods: Voronoi Random Forest Feedback Interface Predictor (VORFFIP)^45^, Consensus Protein-Protein Interaction Site Predictor (cons-PPISP)^46, 47^, Meta Protein-Protein Interaction Site Predictor (meta-PPISP)^48^, and Solvent Accessibility based Protein-Protein Interface Identification and Recognition (SPPIDER)^49^. The consensus predictions of each method were combined to generate the prediction shown in Supplementary Fig. 2. The PyMOL Molecular Graphics System version 1.7 (Schrodinger LLC) was used to visualize the VP24 model and crystal structure.

### Tetracistronic trVLP Assay

This assay was performed essentially as previously described^22, 50^. Briefly, producer HEK 293T cells (5 × 10^5^ cells) were transfected using TransIT-LT1 (according to manufacturer’s directions) with 62.5 ng pCAGGS-NP, 62.5 ng pCAGGS-VP35, 37.5 ng pCAGGS-VP30, 500 ng pCAGGS-L, 125 ng pCAGGS-T7, 5 ng pCAGGS-Luc2, and 125 ng p4cis-vRNA-RLuc, p4cis-vRNA-RLuc-VP24-3x-stop, or p4cis-vRNA-RLuc-VP24-169-176A. Total DNA levels were kept constant by complementing transfections with empty-vector pCAGGS. Twenty-four hours post-transfection, medium was replaced with DMEM containing 5% HI-FBS. Seventy-two hours post-transfection, cell supernatants were harvested, clarified by centrifugation, and transferred to pre-transfected target HEK 293T cells. Cells were lysed in Passive Lysis Buffer (Promega), and Renilla and Firefly luciferase activity was measured using the Dual-Glo Luciferase Assay System (Promega) and a Modulus Microplate luminometer (Turner BioSystems). Twenty-four hours prior to supernatant transfer, target HEK 293T cells (5 × 10^5^ cells) were transfected using TransIT-LT1 with 62.5 ng pCAGGS-NP, 62.5 ng pCAGGS-VP35, 37.5 ng pCAGGS-VP30, 500 ng pCAGGS-L, and 125 ng pCAGGS-TIM1. Twenty-four hours after supernatant transfer to target cells, medium was replaced with DMEM containing 5% HI-FBS. Seventy-two hours post-transfer, cells were lysed in Passive Lysis Buffer, and Renilla and Firefly luciferase activity was measured as above.

### VLP RNA Purification

HEK293 cells (7 × 10^6^ cells) were transfected using TransIT-LT1 (according to manufacturer’s directions) with 875 ng pCAGGS-NP, 875 ng pCAGGS-VP35, 525 ng pCAGGS-VP30, 7000 ng pCAGGS-L, 1750 ng pCAGGS-T7(K179C/M750C), and 1750 ng p4cis-vRNA-RLuc, p4cis-vRNA-RLuc-VP24-3x-stop, p4cis-vRNA-RLuc-VP24-V170A, p4cis-vRNA-RLuc-VP24-N171A, p4cis-vRNA-RLuc-VP24-V170A/N171A, or p4cis-vRNA-RLuc-VP24-169-176A. Twenty-four hours post-transfection, medium was replaced with DMEM containing 5% HI-FBS. Seventy-two hours post-transfection, supernatants were harvested and clarified by centrifugation, 524 × g for 5 min. Clarified supernatants were concentrated using Amicon Ultra-15 Centrifugal Filters with Ultracel-100KDa membranes (Millipore). Following concentration, supernatants were incubated with 500 U benzonase nuclease (Sigma) for 30 min on ice before being mixed with 500 ul phosphate buffered saline (PBS) and purified over a 20% sucrose (w/v in PBS) cushion by ultracentrifugation using an SW21 rotor, 36000 rpm at 4 °C for 2 h. The resulting VLP pellet was lysed in RNA IP (RIP) buffer [150 mM NaCl, 25 mM Tris pH 7.4, 5 mM EDTA, 0.5 mM DTT, 1% Igepal CA-360 supplemented with 100 U/ml RNaseOUT (Thermo Fisher) and cOmplete EDTA-free protease inhibitor cocktail tablets] for 30 min on ice, after which 40% of VLP lysate was combined with 4x Laemmli buffer and 50% of VLP lysate was combined with 3 volumes Trizol LS (Thermo Fisher). Transfected cells were lysed in RIP buffer to generate whole cell lysates (WCL); 45% of the lysate was combined with 4x Laemmli sample buffer and 45% was combined with 3 volumes Trizol LS. RNA was purified and quantified from the Trizol samples as described below. Laemmli sample buffer lysates were subjected to SDS-PAGE/Western blotting as described above.

### RNA Immunoprecipitation

HEK293 cells (2 × 10^6^ cells) were transfected using TransIT-LT1 (according to manufacturer’s directions) with 250 ng pCAGGS-NP-FH, 250 ng pCAGGS-VP35, 500 ng pCAGGS-T7(K179C/M750C), 500 ng p4cis-vRNA-RLuc-VP24-3x-stop, and 500 ng pCAGGS-VP24, 750 ng pCAGGS-VP24 169–176A, 750 ng pCAGGS-VP24 V170A, 750 ng pCAGGS-VP24 N171A, or 750 ng pCAGGS-VP24 V170A/N171A. Twenty-four hours post-transfection, medium was replaced with DMEM containing 5% HI-FBS. Seventy-two hours post-transfection, cells were lysed in RIP buffer and subjected to immunoprecipitation with mouse anti-FLAG M2 antibody or isotype control antibody MOPC21. Immune complexes were precipitated with Dynabeads conjugated to protein G; 15% of the beads were resuspended in 2x Laemmli sample buffer, while the remainder was resuspended in Trizol (Thermo Fisher). One portion (15%) of pre-immune whole cell lysate was reserved and subjected to acetone precipitation; precipitated protein was resuspended in 2x Laemmli sample buffer. Another portion (5%) of pre-immune whole cell lysate was reserved and combined with three volumes Trizol LS. RNA was purified and quantified from the Trizol LS samples as described below. Laemmli sample buffer lysates were subjected to SDS-PAGE/Western blotting as described above.

### RNA Purification and Quantification

RNA was purified from all Trizol samples using the Direct-zol RNA MiniPrep Kit (Zymo Research) with in-column DNAse treatment according to manufacturer’s directions. Each RNA sample was eluted in 50 ul water.

EBOV minigenome RNA was quantified via quantitative reverse transcription PCR (RT-qPCR) on the Rotor-Gene 6000 thermal cycler (Qiagen) using the QuantiFast Probe PCR Kit (Qiagen) according to manufacturer’s directions. Each reaction used 5 ul of RNA and 0.4 uM and 0.2 uM of primers and probe (TIB Molbiol), respectively. The primer/probe sequences were as follows: 5′-TTTCCAATCCAAGTCACACTG-3′ (forward primer), 5′-ATTGCACATACTTTTTGCCC-3′ (reverse primer), and 5′-TCCTCAGATGTAAGCATGCAGGCAA-3′ (probe with 5′ 6FAM fluorophore and 3′ BlackBerry (BBQ) quencher). Cycling conditions were as follows: 10 min initial reverse transcription at 50 °C, 5 min initial denaturation/activation at 95 °C, followed by 40 cycles of 5 sec denaturation at 95 °C and 10 sec annealing/extension at 60 °C. Data were acquired at the end of the annealing/extension step in the green (510 nm) channel.

A standard curve was used to quantify the amount of EBOV minigenome RNA in picograms. Briefly, p4cis-vRNA-RLuc-VP24-3x-stop was linearized using the BlpI restriction endonuclease (New England Biolabs) before being subjected to *in vitro* transcription using the MEGAscript T7 Transcription Kit (Thermo Fisher) according to manufacturer’s directions. Transcribed RNA was purified using the MEGAclear Kit (Thermo Fisher), according to manufacturer’s directions, and RNA concentration was determined with a NanoDrop spectrophotometer. A set of five RNA standard samples ranging in concentration from 0.0001–1 pg was created by ten-fold serial dilution and included in every RT-qPCR reaction, providing sufficient range to generate a standard curve and interpolate or extrapolate the concentration of RNA in all test samples using the Rotor-Gene 6000 Series software v1.7 (Qiagen).

Total cellular RNA was quantified with the Low Abundance RNA Quantification Kit (Norgen Biotek Corp.), according to manufacturer’s directions, using the CFX96 Real-Time System (Bio-Rad).

### Minigenome Assay

This assay was performed essentially as previously described^27, 51^. Briefly, HEK 293 cells (1 × 10^6^ cells) were transfected using TransIT-LT1 (according to manufacturer’s directions) with 125 ng pCAGGS-NP, 125 ng pCAGGS-VP35, 75 ng pCAGGS-VP30, 1000 ng pCAGGS-L, 250 ng pCAGGS-T7, 250 ng p1cis-vRNA-RLuc (monocistronic minigenome), 100 ng pCAGGS-Luc2 (encoding Firefly luciferase as a transfection control), and pCAGGS-VP24 WT or 169–176A. Total DNA levels were kept constant by complementing transfections with empty-vector pCAGGS. Forty-eight hours post-transfection, cells were harvested and lysed in Passive Lysis Buffer, and Renilla and Firefly luciferase activity was measured using the Dual-Glo Luciferase Assay System and a Modulus Microplate luminometer. Data are presented as relative light units (RLU) on a log scale with the Renilla luciferase signal normalized against the average of the Firefly luciferase signal. A portion of whole cell lysate was reserved and subjected to acetone precipitation; precipitated protein was re-suspended in 2x Laemmli sample buffer and subjected to SDS-PAGE/Western blotting as described above.

### Statistical Analyses

Data in Figs 3–5, as well as Supplementary Fig. 5, are expressed as mean values, plus or minus the standard error of the mean (SEM). Statistical comparisons of the data in Figs 3, 4 and 5 and Supplementary Fig. 5 were performed using an ordinary one-way ANOVA test and Dunnett’s multiple comparisons test using Prism (version 7) software. *p*-values less than or equal to 0.05 were marked with one asterisk (*), less than or equal to 0.01 were marked with two asterisks (**), less than or equal to 0.001 were marked with three asterisks (***), and less than or equal to 0.0001 were marked with four asterisks (****).

### Data Availability

The data generated and/or analyzed during the current study are available from the corresponding author on reasonable request.

## Electronic supplementary material

Supplementary Figures 1–9
