# Supplementary material for: Ebola virus VP24 interacts with NP to facilitate nucleocapsid assembly and genome packaging
Source: Sci Rep. 2017 Aug 9;7:7698. doi: 10.1038/s41598-017-08167-8 (PMC5550494; doi:10.1038/s41598-017-08167-8)
Supplement: Supplementary file 1 — Supplementary Figures 1–9 [file 41598_2017_8167_MOESM1_ESM.pdf]

SUPPLEMENTARY DATA AND FIGURE LEGENDS

for

Ebola virus VP24 interacts with NP to facilitate nucleocapsid assembly and genome packaging

Logan Banadyga, Thomas Hoenen, Xavier Ambroggio, Eric Dunham, Allison Groseth, and Hideki Ebihara

SUPP. FIG. 1

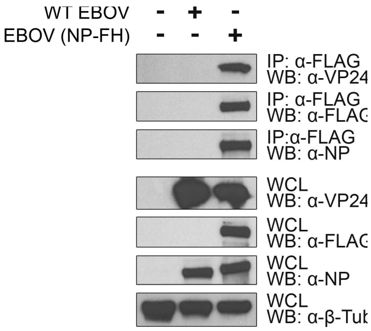

WT EBOV - + -
EBOV (NP-FH) - - +
IP: α-FLAG
WB: α-VP24
IP: α-FLAG
WB: α-FLAG
IP:α-FLAG
WB: α-NP
WCL
WB: α-VP24
WCL
WB: α-FLAG
WCL
WB: α-NP
WCL
WB: α-β-Tub

Supplementary Figure 1 | NP and VP24 interact during EBOV infection. Vero E6 cells were infected at MOI 1 with either wild-type (WT) EBOV or recombinant EBOV expressing NP-FH (EBOV NP-FH). Four days post-infection, cells were lysed and immunoprecipitated with mouse anti-FLAG or isotype control (Iso) antibodies. Immunoprecipitation (IP) and whole cell lysate (WCL) fractions were subjected to Western blot (WB) with mouse anti-FLAG, mouse anti-NP 74-7, rabbit anti-VP24, or rabbit anti-β-tubulin antibodies.

SUPP. FIG. 2

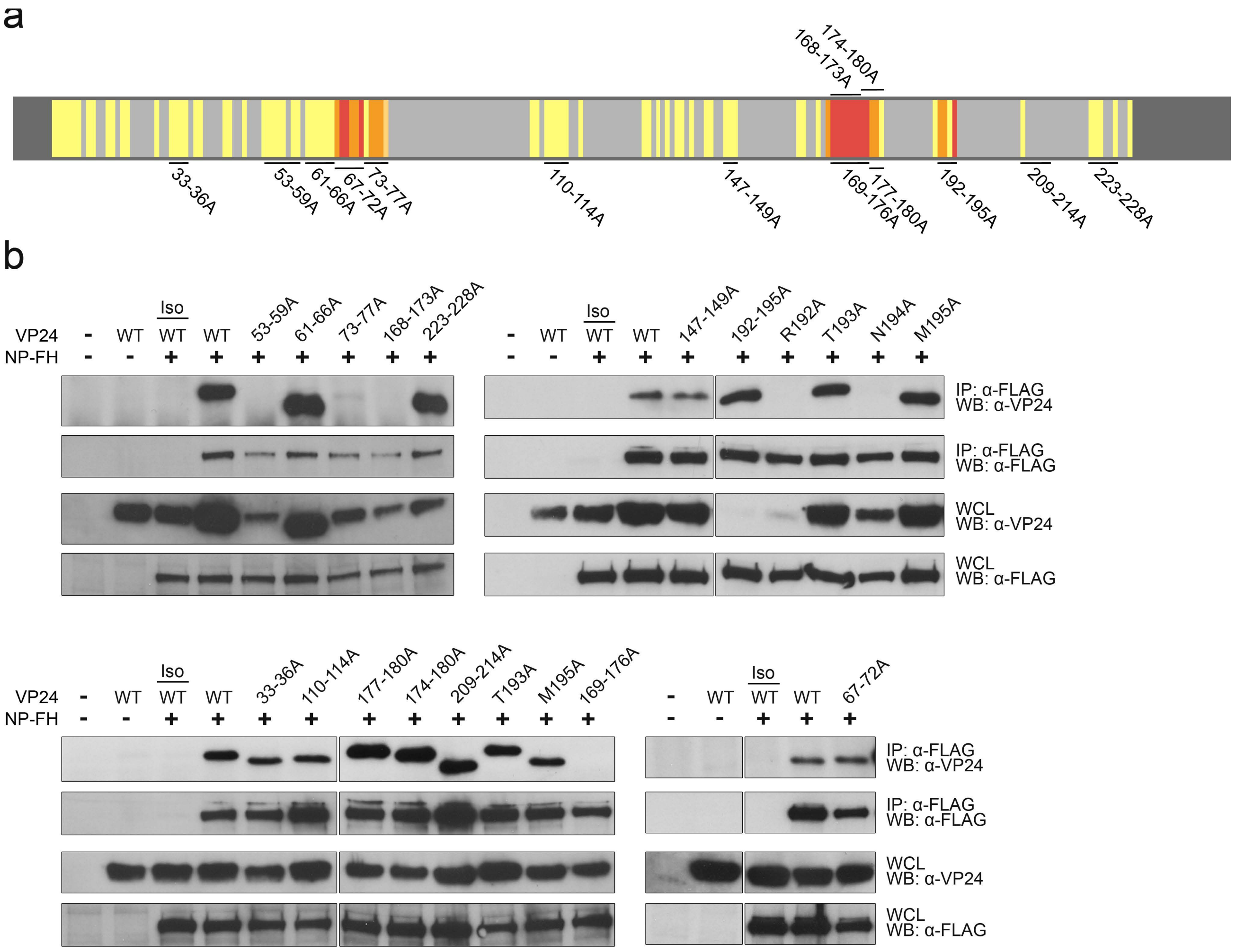
a
174-180A
168-173A
33-36A
53-59A
61-66A
67-72A
73-77A
110-114A
147-149A
169-176A
177-180A
192-195A
209-214A
223-228A
b
VP24
NP-FH
Iso
WT
53-59A
61-66A
73-77A
168-173A
223-228A
147-149A
192-195A
R192A
T193A
N194A
M195A
IP: α-FLAG
WB: α-VP24
IP: α-FLAG
WB: α-FLAG
WCL
WB: α-VP24
WCL
WB: α-FLAG
33-36A
110-114A
177-180A
174-180A
209-214A
169-176A
67-72A

Supplementary Figure 2 | VP24 mutants interact with NP. (**a**) The consensus results of four protein-protein interaction prediction algorithms for our VP24 structural model are displayed on a linear diagram of VP24. Regions are colored red, dark orange, light orange, and yellow according to a decrease in prediction consensus that these amino acids would be involved in protein-protein interactions. Regions colored light grey were not predicted to be involved in interactions, and regions colored in dark grey were not part of our VP24 structural model. The locations of all VP24 alanine mutants are indicated. (**b**) HEK 293 cells were co-transfected with pCAGGS-NP-FH and pCAGGS-Wild-type (WT) VP24 or the indicated VP24 alanine mutant. Lysates were immunoprecipitated with mouse anti-FLAG or isotype control (Iso) antibodies, and immunoprecipitation (IP) and whole cell lysate (WCL) fractions were subjected to Western blot (WB) with mouse anti-FLAG or rabbit anti-VP24 antibodies.

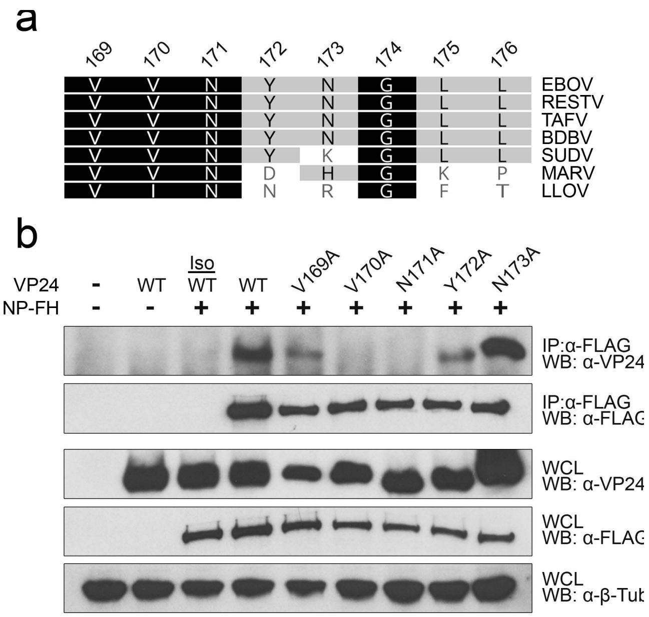
a
169
170
171
172
173
174
175
176
V V N Y N G L L EBOV
V V N Y N G L L RESTV
V V N Y N G L L TAFV
V V N Y N G L L BDBV
V V N Y K G L L SUDV
V V N D H G K P MARV
V I N N R G F T LLOV
b
VP24 - WT Iso WT WT V169A V170A N171A Y172A N173A
NP-FH - - + + + + + + +
IP:α-FLAG
WB: α-VP24
IP:α-FLAG
WB: α-FLAG
WCL
WB: α-VP24
WCL
WB: α-FLAG
WCL
WB: α-β-Tub

Supplementary Figure 3 | VP24 V170 and N171, but not V169, Y172, or N173, are critical for interacting with NP. (**a**) A sequence alignment for amino acids 169-176 of VP24 from representative sequences of all filoviruses. The multiple sequence alignment was generated using the Geneious algorithm as implemented in Geneious v9.1.5 and is highlighted according to amino acid sequence conservation, with black indicating identical or highly similar amino acids, grey indicating similar amino acids, and white indicating dissimilar amino acids. The VP24 amino acid sequences used were as follows (with GenBank accession numbers indicated in parentheses): EBOV, Ebola Virus (AAD14588.1); RESTV, Reston Virus (AAN04453.1); TAFV, Tai Forest Virus (ACI28635.1); BDBV, Bundibugyo Virus (AGL73476.1); SUDV, Sudan Virus (AAU43889.1); MARV, Marburg Virus (ABE27066.1); and LLOV, Lloviu Virus (AER23678.1). (**b**) HEK 293 cells were co-transfected with pCAGGS-NP-FH and pCAGGS-Wild-type (WT) VP24 or a VP24 point mutant: pCAGGS-VP24 V169A, pCAGGS-VP24 V170A, pCAGGS-VP24 N171A, pCAGGS-VP24 Y172A, or pCAGGS-VP24 N173A. Lysates were immunoprecipitated with mouse anti-FLAG or isotype control (Iso) antibodies, and immunoprecipitation (IP) and whole cell lysate (WCL) fractions were subjected to Western blot (WB) with mouse anti-FLAG, rabbit anti-VP24, or rabbit anti-β-tubulin antibodies.

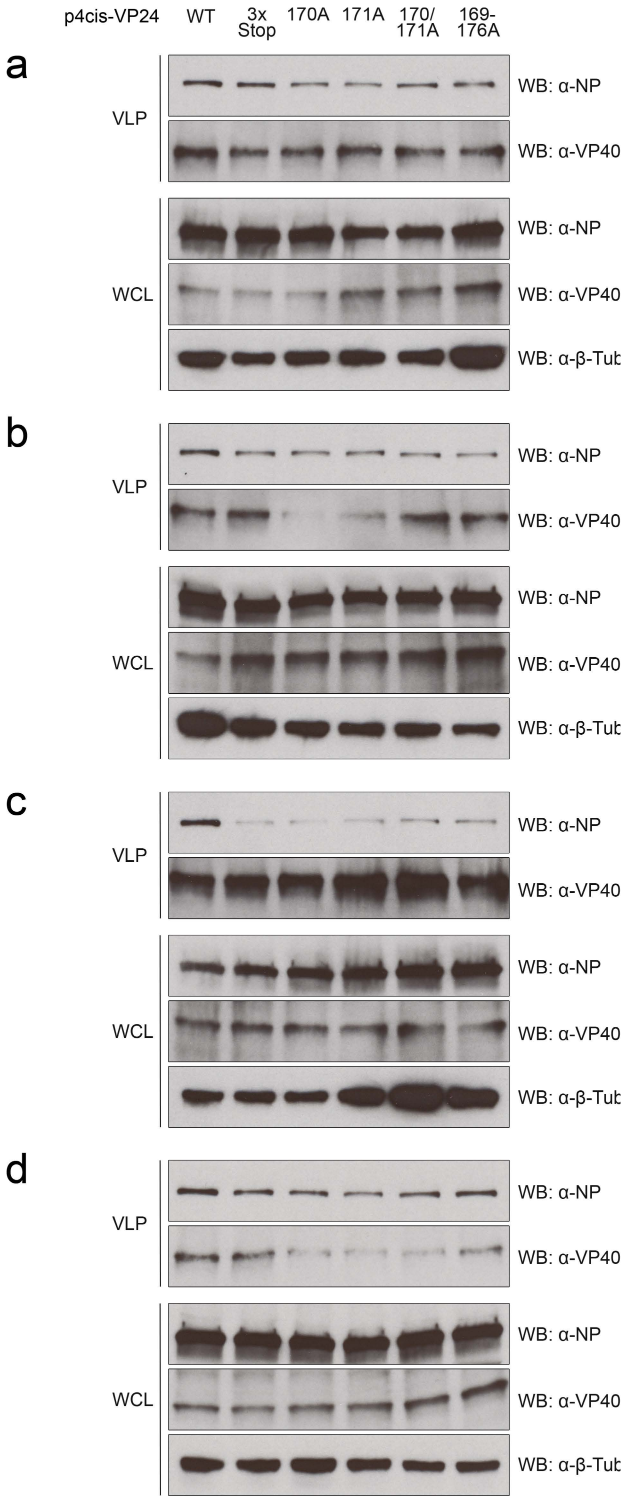
p4cis-VP24
WT
3x Stop
170A
171A
170/ 171A
169- 176A
a
b
c
d
VLP
WCL
WB: α-NP
WB: α-VP40
WB: α-β-Tub

Supplementary Figure 4 | NP Incorporation into VLPs is reduced in the absence of wild-type VP24. (**a-d**) HEK 293 cells were co-transfected and VLPs were harvested and purified as described for Figure 4. VLP lysates and producer cell whole cell lysates (WCL) from the Figure 4 experiment were subjected to Western blot (WB) with mouse anti-NP, rabbit anti-VP40, or rabbit anti-β-tubulin antibodies. The results from four independent experiments are shown (**a-d**). The VLP samples and anti-β-tubulin WBs are also displayed in Fig. 4d.

Supp Fig. 5

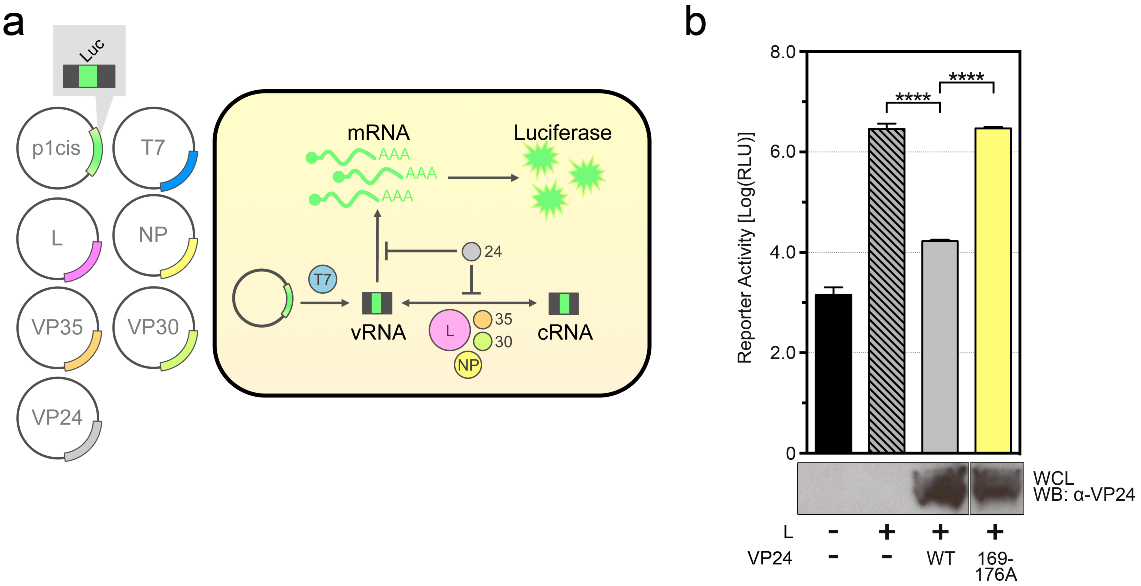
a
Luc
p1cis
T7
L
NP
VP35
VP30
VP24
mRNA
AAA
AAA
AAA
Luciferase
24
T7
vRNA
cRNA
L
35
30
NP
b
8.0
6.0
4.0
2.0
0
Reporter Activity [Log(RLU)]
****
****
WCL
WB: α-VP24
L
-
+
+
+
VP24
-
-
WT
169-
176A

Supplementary Figure 5 | The VP24-NP interaction is necessary to inhibit EBOV minigenome activity. (**a**) A schematic of the EBOV minigenome system. The EBOV minigenome system recapitulates the viral RNA polymerase complex in transfected cells and permits replication and transcription of a monocistronic minigenome (p1cis) encoding the reporter protein Renilla luciferase. Initial replication of p1cis by the T7 polymerase produces a viral RNA (vRNA) template, which is then replicated, via complementary RNA (cRNA) intermediates, by the EBOV ribonucleoprotein complex, consisting of L, NP, VP35, and VP30. From the minigenome, the ribonucleoprotein complex also transcribes Renilla luciferase (Luc) mRNA, which, when translated by host cellular machinery, produces a measurable signal proportional to the degree of EBOV minigenome replication and transcription. Notably, over expression of Wild-type VP24 inhibits minigenome replication and transcription, thereby reducing Renilla luciferase signal. (**b**) HEK 293 cells were co-transfected with p1cis-vRNA-RLuc (p1cis), as well as pCAGGS-luc2, encoding Firefly luciferase as a transfection control, pCAGGS-T7, pCAGGS-L, pCAGGS-NP, pCAGGS-VP35, pCAGGS-VP30, and pCAGGS-Wild-type (WT) VP24 or pCAGGS-VP24 169-176A. Data are presented as relative light units (RLU) on a log scale with the Renilla luciferase signal normalized against the average of a control Firefly luciferase signal. The means and standard error of the mean for 3 independent experiments are shown (****, $p \leq 0.0001$). In the absence of the polymerase L, no minigenome replication/transcription is possible and luciferase reporter activity was low. Conversely, with an intact polymerase complex, but in the absence of VP24, reporter activity was over three logs higher, indicating robust replication/transcription. The presence of WT VP24, as previously reported, inhibited replication/transcription, and luciferase reporter levels were significantly decreased. Conversely, VP24 169-176A was unable to inhibit minigenome replication/transcription. Whole cell lysates (WCL) were subjected to Western blot (WB) with rabbit anti-VP24 antibody, demonstrating that expression levels of WT VP24 and VP24 169-176A were comparable.

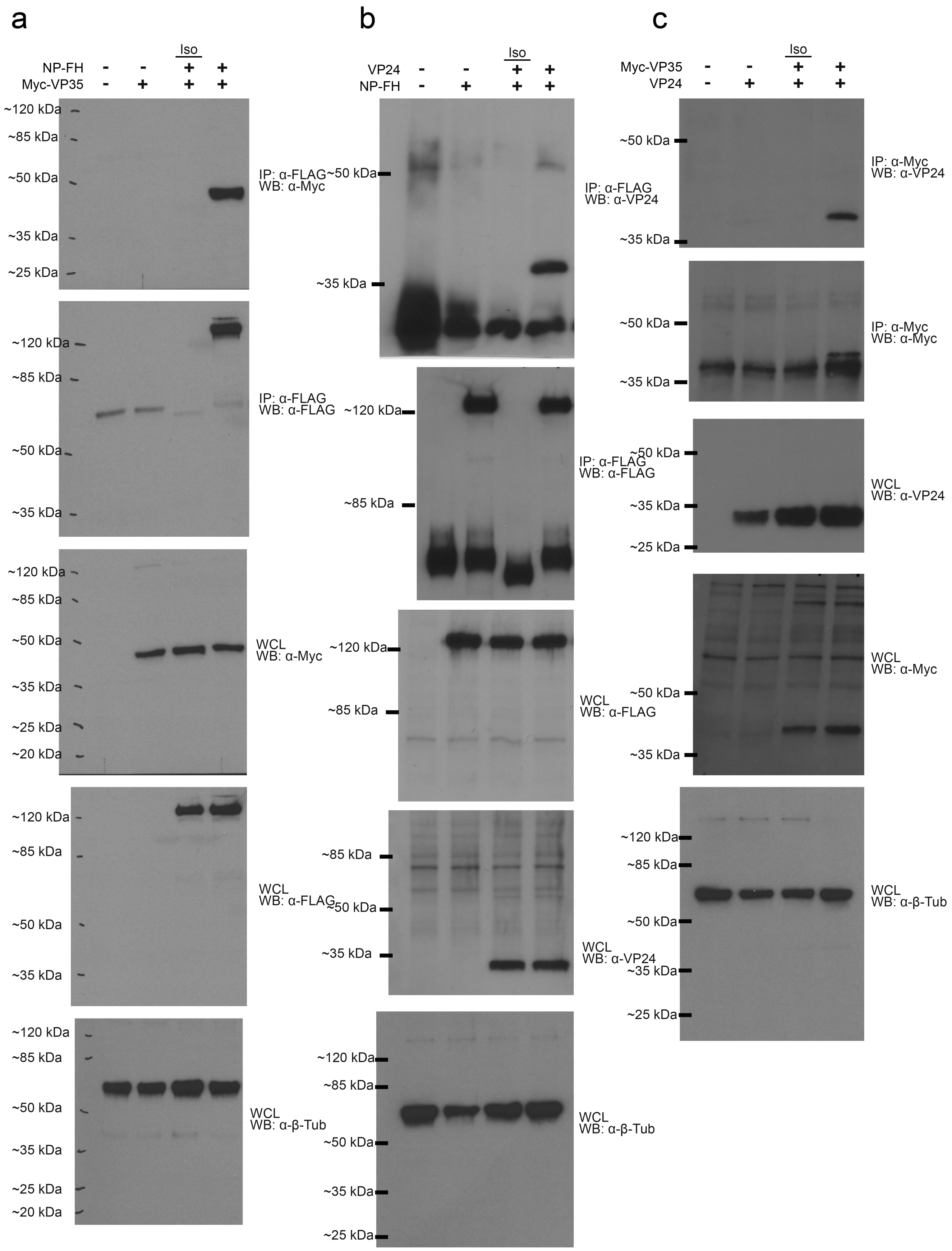
a
Iso
NP-FH - - + +
Myc-VP35 - + + +
~120 kDa
~85 kDa
~50 kDa
~35 kDa
~25 kDa
IP: α-FLAG
WB: α-Myc
IP: α-FLAG
WB: α-FLAG
WCL
WB: α-Myc
WCL
WB: α-FLAG
WCL
WB: α-β-Tub
~20 kDa
b
Iso
VP24 - - + +
NP-FH - + + +
IP: α-FLAG
WB: α-VP24
IP: α-FLAG
WB: α-FLAG
WCL
WB: α-FLAG
WCL
WB: α-VP24
WCL
WB: α-β-Tub
c
Iso
Myc-VP35 - - + +
VP24 - + + +
IP: α-Myc
WB: α-VP24
IP: α-Myc
WB: α-Myc
WCL
WB: α-VP24
WCL
WB: α-Myc
WCL
WB: α-β-Tub

Supplementary Figure 6 | NP, VP35, and VP24 interact independently with each other. Western blots in Figure 1 were cropped for presentation; they are presented here in full-length. (**a-c**) HEK 293 cells were co-transfected with pCAGGS-NP-FH and pCAGGS-Myc-VP35 (**a**), pCAGGS-NP-FH and pCAGGS-VP24 (**b**), or pCAGGS-Myc-VP35 and pCAGGS-VP24 (**c**). Lysates were immunoprecipitated with mouse anti-FLAG (**a**, **b**), mouse anti-Myc (**c**), or isotype control (Iso; **a-b**) antibodies, and immunoprecipitation (IP) and whole cell lysate (WCL) fractions were subjected to Western blot (WB) with mouse anti-FLAG, mouse anti-Myc, rabbit anti-VP24, and rabbit anti-β-tubulin antibodies. The precipitated proteins in the IP fraction are labeled with arrowheads, and the light chain (LC) of the mouse anti-Myc antibody is indicated with an arrow (**a-b**). IP/Western blot data are representative of at least three independent experiments.

Supp Fig. 7

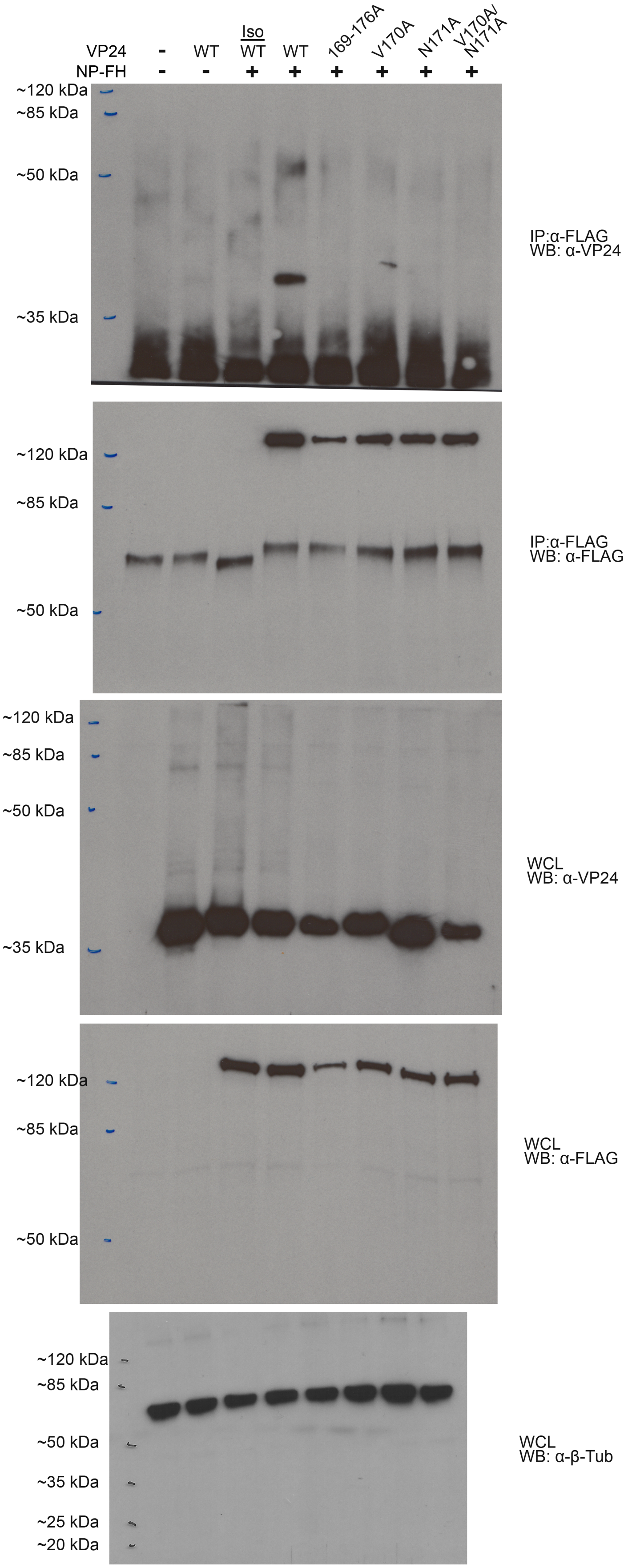
VP24
NP-FH
-
WT
Iso WT
WT
169-176A
V170A
N171A
V170A/ N171A
~120 kDa
~85 kDa
~50 kDa
~35 kDa
~25 kDa
~20 kDa
IP:α-FLAG
WB: α-VP24
IP:α-FLAG
WB: α-FLAG
WCL
WB: α-VP24
WCL
WB: α-FLAG
WCL
WB: α-β-Tub

Supplementary Figure 7 | VP24 amino acids V170 and N171 are critical for interacting with NP. Western blots in Figure 2 were cropped for presentation; they are presented here in full-length. HEK 293 cells were co-transfected with pCAGGS-NP-FH and pCAGGS-Wild-type (WT) VP24 or a VP24 point mutant: pCAGGS-VP24 169-176A, pCAGGS-VP24 V170A, pCAGGS-VP24 N171A, or pCAGGS-VP24 V170A/N171A. Lysates were immunoprecipitated with mouse anti-FLAG or isotype control (Iso) antibodies, and immunoprecipitation (IP) and whole cell lysate (WCL) fractions were subjected to Western blot (WB) with mouse anti-FLAG, rabbit anti-VP24, or rabbit anti-β-tubulin antibodies. IP/Western blot data are representative of at least three independent experiments.

Supp Fig. 8

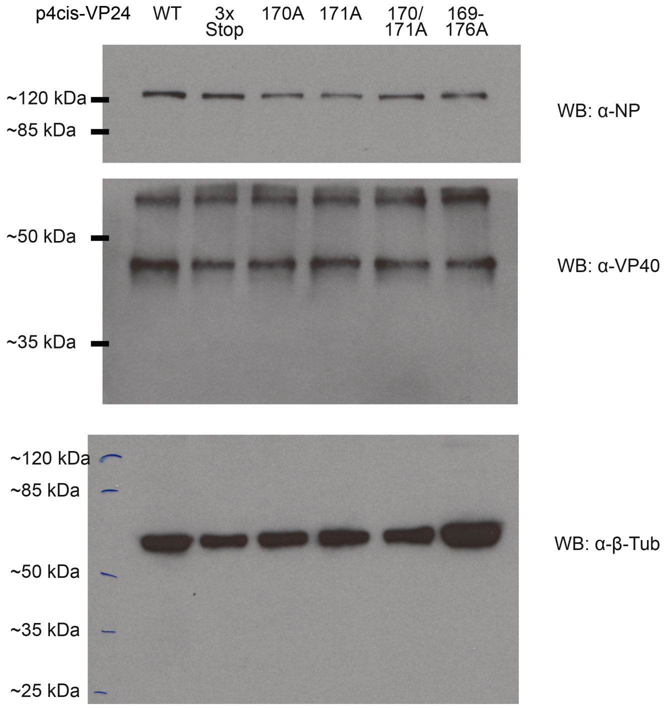

p4cis-VP24
WT
3x Stop
170A
171A
170/ 171A
169- 176A
~120 kDa
~85 kDa
WB: α-NP
~50 kDa
~35 kDa
WB: α-VP40
~120 kDa
~85 kDa
~50 kDa
~35 kDa
~25 kDa
WB: α-β-Tub

Supplementary Figure 8 | VLPs produced with mutant VP24 contain reduced levels of EBOV minigenome. Western blots in Figure 4 were cropped for presentation; they are presented here in full-length. VLP lysates and producer cell whole cell lysates (WCL) were subjected to Western blot (WB) with mouse anti-NP, rabbit anti-VP40, and rabbit anti-β-tubulin antibodies.

Supp Fig. 9

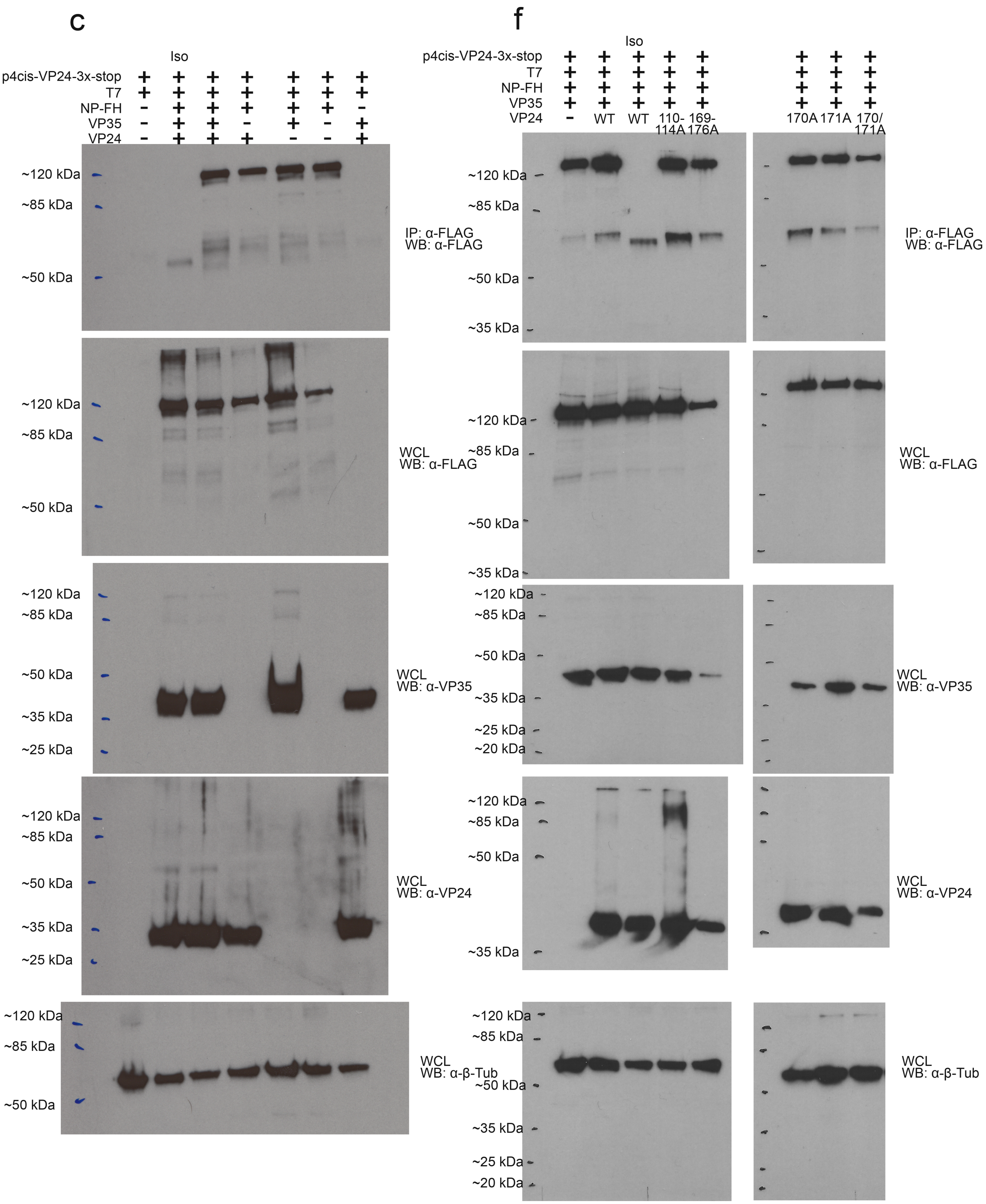
c
Iso
p4cis-VP24-3x-stop
T7
NP-FH
VP35
VP24
~120 kDa
~85 kDa
~50 kDa
~35 kDa
~25 kDa
IP: α-FLAG
WB: α-FLAG
WCL
WB: α-FLAG
WCL
WB: α-VP35
WCL
WB: α-VP24
WCL
WB: α-β-Tub
f
Iso
p4cis-VP24-3x-stop
T7
NP-FH
VP35
VP24
WT
WT
110-114A
169-176A
170A
171A
170/171A
~20 kDa

Supplementary Figure 9 | VP24 is critical for EBOV genome encapsidation. Western blots in Figure 5 were cropped for presentation; they are presented here in full-length. Immunoprecipitation (IP) and whole cell lysate (WCL) fractions were subjected to Western Blot (WB) with mouse anti-FLAG, mouse anti-VP35, rabbit anti-VP24, or rabbit anti-β-tubulin antibodies (**c**, **f**). Note that VP24 mutant 110-114A was not presented in Figure 5.
